# Lifecycle model-based evaluation of infant 4CMenB vaccination in the UK

**DOI:** 10.1007/s10198-023-01654-y

**Published:** 2024-01-05

**Authors:** J. P. Sevilla, Daniel Tortorice, David Kantor, John Regan, Kinga H. Meszaros, Ekkehard C. Beck, Najida Begum, David E. Bloom

**Affiliations:** 1 Data for Decisions (DfD) LLC, 681 Main Street, Suite 3-37, Waltham, MA 02457 USA; 2 GSK, Value Evidence, Wavre, Belgium; 3 Freelance C/O GSK, Wavre, Belgium

**Keywords:** Lifecycle model, Health augmented, Cost–benefit analysis, 4CMenB, I1, H51, H43

## Abstract

**Objectives:**

Invasive meningococcal disease, an uncommon but severe disease, imposes catastrophic health and economic burdens. Cost–utility analysis (CUA) assumes separability in lifetime health and economic variables and cannot capture the full value of preventing such burdens. We overcome these limitations with a retrospective societal perspective cost–benefit analysis (CBA) of meningococcal serogroup B vaccination (4CMenB) of one infant cohort in the United Kingdom using a health-augmented lifecycle model (HALM) incorporating health’s interactions with consumption, earnings, non-market time and financial risk.

**Methods:**

We used a static Markov model of vaccination’s health impact and an HALM to estimate the private willingness to pay (PWTP) for the intrinsic and instrumental value of health under perfect capital markets, financial risk protection in the absence of insurance against permanent disability, parental spillovers, and acute phase disability. We estimated social WTP (SWTP) incorporating social severity preferences. We estimated rates of return that inform health payer reimbursement decisions, finance ministry budgeting decisions, and legislature taxation decisions. An expert Advisory Board investigated the validity of applying the HALM to infant 4CMenB.

**Results:**

The PWTP for a 2 + 1 vaccination schedule is £395, comprising £166 of disability insurance value, £79 of positive parental spillover value, £28 in the value of averting acute phase disability, and £122 in residual intrinsic and instrumental value of health. SWTP is £969.

**Conclusions:**

HALM-based CBA provides an empirically richer, more utility–theoretically grounded approach to vaccine evaluation than CUA, demonstrating good value for money for legislatures (based on private values) and for all decision-makers (based on social values).

**Supplementary Information:**

The online version contains supplementary material available at 10.1007/s10198-023-01654-y.

## Introduction

Invasive meningococcal disease (IMD), an uncommon but severe disease, affects mostly infants to young adults. Serogroup distributions vary globally [1] but meningococcal serogroup B (MenB) is most prevalent in Europe and the United States (US) [2]. IMD progresses rapidly and has a high acute phase mortality. In England, from 2008 to 2015, patients’ risk of death across all ages was 7.4%, and 85% of deaths occurred within a day of diagnosis [3]. Survivors risk life-long severe health and socioeconomic consequences, which in turn affect families, caregivers, and wider society [4–6]. Despite its low incidence (in 2011/2012 in England and Wales, there were 25 IMD cases per 100,000 infants [7]), the severity of IMD has created significant public concern [8], leading to the United Kingdom’s (UK) introduction of meningococcal conjugate vaccine against serogroup C in 1999 (and later a quadrivalent vaccine against A, C, W and Y) for young adolescents and meningococcal B vaccination (4CMenB, Bexsero) in 2015 for infants.

The infant 4CMenB vaccine was particularly controversial. Immunizations are typically included in the UK National Health Service (NHS) immunization schedule following recommendations for such inclusion by the UK Joint Committee on Vaccinations and Immunizations (JCVI). JCVI recommendations, in turn, depend in part on outcomes of a cost-effectiveness analysis of cost-effectiveness (CEA) [9]. In 2013, JCVI issued an interim statement concluding that infant 4CMenB vaccination was “unlikely to [be cost-effective] at any price” [10], such cost-ineffectiveness in part reflecting MenB’s low incidence [11]. This statement was met by widespread protest, including from experts, the Meningitis Now charity, and shadow ministers [12], citing the severity of its impacts [13]. These protests led JCVI to consult with stakeholders and to conduct a revised CEA that included a broader range of vaccine benefits and revised model assumptions. These added benefits included vaccine-averted litigation costs and parental quality-of-life losses [10]. An important element of the revised analysis was the assumption, no doubt informed by the protests, that society attributed extra value to preventing severe over mild disease. They modeled this societal value through a quality-of-life adjustment factor (QAF) that multiplies long-term QALY losses from MenB (and therefore long-term 4CMenB-related QALY gains) by 3 [14]. Thus, a QALY gained from preventing severe long-term disease was valued three times as much as a QALY gained from preventing mild disease. The revised CEA led JCVI to conclude that infant 4CMenB could be cost-effective at a low enough price, and to recommend its inclusion in the NHS immunization schedule [10]. This led to the UK being the first country to offer 4CMenB in its infant immunization schedule [15].

Value-for-money (VfM) assessments of vaccination programs typically inform health payer (e.g., the UK Department of Health and Social Care (DHSC)) decisions regarding National Vaccine Introduction (NVI). Payers with fixed budgets should introduce a vaccine only if its VfM exceeds that of health technologies likely to be displaced by it. VfM assessments can and should also inform decisions by finance ministries and legislatures, like the UK Treasury and Parliament [16]. If a vaccine has high VfM relative to other publicly financed non-health expenditures, then finance ministries can expand health budgets to accommodate it without displacing other health technologies. If a vaccine has high VfM relative to the opportunity cost of household funds, then legislatures can raise taxes to accommodate the vaccine without displacing other public spending [17].

Two issues in VfM assessment are the choices between health payer and societal perspectives, and between cost–utility and cost–benefit analysis (CUA and CBA). The payer perspective values only a technology’s health gains and payer budget consequences, allocates budgets to maximize health, and reimburses a technology if its incremental cost-effectiveness ratio (ICER) falls below the finance ministry’s (“policymaker”) willingness to pay (WTP) for health, as reflected in the marginal funded health technology’s ICER. This perspective assumes optimality of the payer’s budget and does not aim to inform finance ministry and legislative decisions. The societal perspective, in contrast, considers the broader socioeconomic impacts of health, and values health and such impacts at individuals’ WTP for them. It does not assume optimality of the payer’s budget and can inform finance ministries and legislative decisions affecting that budget.

CUA assumes every quality-adjusted life year (QALY) has equal value (“a QALY is a QALY is a QALY”). It can be used within both payer and societal perspectives, where that value equals the policymaker’s WTP (e.g., in the UK, estimated at 50% of per capita gross domestic product [PCGDP]) [17] and individual’s WTP (benchmarked at 1–3 times PCGDP) [18, 19] per QALY, respectively. But within a societal perspective, CUA’s equal-value-per-QALY assumption lacks robust utility-theoretic or empirical justification [20, 21]. A utility-maximizing individual has constant WTP per QALY only if consumption and non-market time are constant throughout life and capital markets are perfect (discussed further below) [20, 21], which are clearly false. The benchmark individual WTP of one to three times PCGDP also has been criticized as having uncertain economic justification and policy relevance [18, 22].

CBA, in contrast, assumes every currency unit of an individual’s WTP has equal value, which allows equal quantities of health to have different monetary values depending on their differential socioeconomic implications. Since utility theory and economic evidence—e.g., age-varying consumption and non-market time, imperfect capital markets (ICM)—suggest such differential implications, CBA may have stronger economic justification than CUA. CBA’s equal-value-per-pound assumption problematically gives greater weight to the preferences of the wealthy, who have greater ability to pay (ATP). The pragmatic solution to this, which we took, is to focus on a representative individual, thus eliminating ATP differentials. A longer-term solution is to generalize CBA using social welfare functions (SWF) [23], which allows for ATP adjustments and priority for the worse off.

Many past evaluations of 4CMenB used payer perspective CUA and yielded low VfM estimates or high ICERs because they inadequately accounted for IMD’s severe health and socioeconomic burdens [11, 24–32]. Exceptions [26, 33] include Beck et al. [34], who found infant 4CMenB to be cost-effective in the UK when a societal perspective and broader health and socioeconomic benefits are considered. We evaluated infant 4CMenB with a societal perspective CBA using a health-augmented lifecycle model (HALM) to generate expressions for individual WTP for health. The HALM is a traditional microeconomic lifetime utility maximization model, augmented to incorporate mortality and morbidity risks, at whose optimal solution we can derive individual WTP for changes in the probability of being in one or another age-specific health state, and which WTP reflects the impact of disability on lifecycle economic quantities like wages, earnings, consumption, and financial risk protection.

Using the HALM has two advantages, one is scientific, the other decision theoretic. First, the HALM flexibly and comprehensively incorporates the complex interactions over the lifecycle and across health states between health and a full range of important economic quantities like consumption, paid and unpaid work, leisure, and financial risks. Variations in such interactions across age and health states refute the equal-value-per-QALY CUA assumption since health associated with more net economic goods generates higher value. Second, the HALM fits within a value framework (utility theory) with attractive axiomatic and ethical foundations (the expected utility axioms and welfarism) capable of informing policy decisions. Instead of ICERs, we used a VfM indicator, the rate of return (RoR) [16, 35], that informs payer, finance ministry, and legislative decisions. Unlike CUA, our HALM-derived VfM formulas have utility-theoretic foundations more consistent with welfare economics and economic theory and evidence.

## Methods

We retrospectively evaluated introducing infant 4CMenB vaccination in the UK in 2015 using a societal perspective CBA. We compared vaccinating a single birth cohort (at 2, 4 and 12 months using a 2 + 1 schedule) in 2015 relative to no vaccination and followed this cohort over a 100-year modeling horizon [34]. Our analysis depended on two models: a Markov disease model that determines the probabilities of different health states over the lifetime, and a HALM that quantifies the value of those health states. Vaccination reduces disease incidence but not severity. We provide high-level summaries of methods and data here. The Supplementary Appendix contains more details on model assumptions, methods, inputs, and calibration. It also includes mathematical derivations and a full set of input data tables. The model was programmed in Python.

### Disease model

#### Markov model

**Fig. 1:**
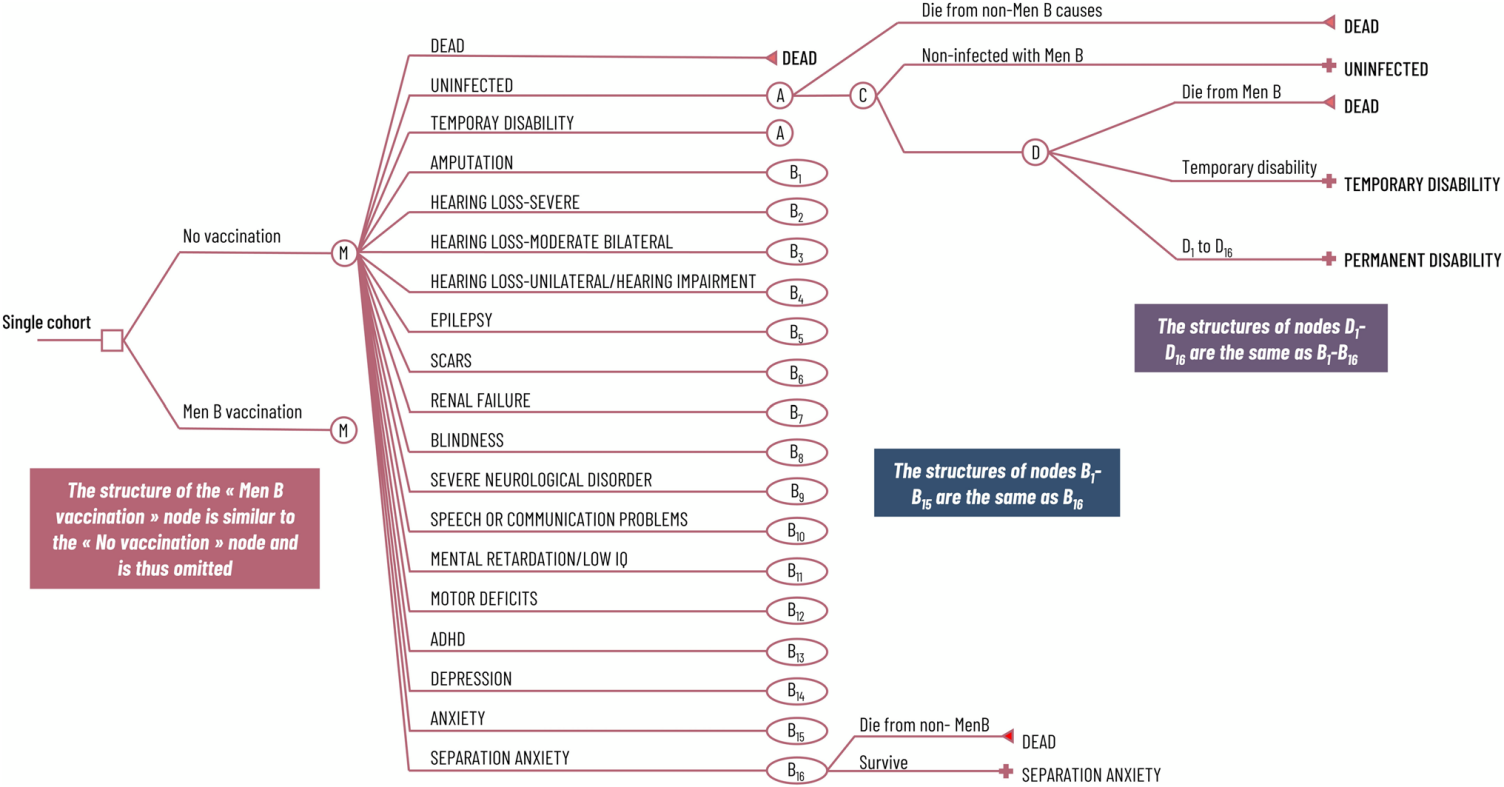
Markov model of disease to quantify vaccination’s effect on mortality and morbidity risk over an individual’s lifetime. The cohort enters the annual Markov cycle tree at node “M.” The cohort then faces a sequence of risks: at node A, the risk of dying from non-MenB-related causes, driven by survivorship values from life tables; at node C, the probability of Men-B infection, driven by the MenB incidence rate; and at node D the risks of death and disability from infection, where the risk of death is given by the case fatality rate, and the risk of various kinds of MenB-related disabilities are driven by prevalence of such disabilities among survivors. An individual who gets temporary disability (${\text{j}}=3)$ from MenB achieves perfect recovery within the same year and faces the same prospects as an individual in the uninfected state. The structure of the nodes B_1_–B_15_ are same as that of node B_16_ denote all permanent disabilities (i.e., sequelae from MenB). To derive node B_1_, replace “SEPARATION ANXIETY” with “AMPUTATION” at all places. *ADHD* attention deficit hyperactivity disorder, *IQ* intelligence intelligent quotient, *MenB* meningococcal serogroup B

Our Markov model has 19 states indexed by $j$, where $j=1$ represents death, $j=2$ the uninfected state, $j=3$ a temporary disability state, and $j=4,\dots ,19$ various permanent physical, neurological, and psychological disability states. We allowed 100 annual cycles indexed by age $i=0,\dots ,99$, with cycle graphs presented in Fig. 1. The uninfected birth cohort faces a sequence of risks: at node A, the risk of dying from non-MenB-related causes; at node C, the probability of MenB infection, driven by MenB incidence and vaccination status; and at node D, the risks of death and disability from infection, driven by the case fatality rate (CFR) and disability prevalences among survivors [4, 36].

The temporarily disabled fully recover within the same cycle and face the same subsequent risks as the uninfected. For simplicity and conservatively [37], the permanently disabled face no risks of elevated mortality, reinfection, or multiple sequelae. Death is the only absorbing state. The disease model generated age-specific probabilities of each health state with and without vaccination.

#### Parameters

**Fig. 2:**
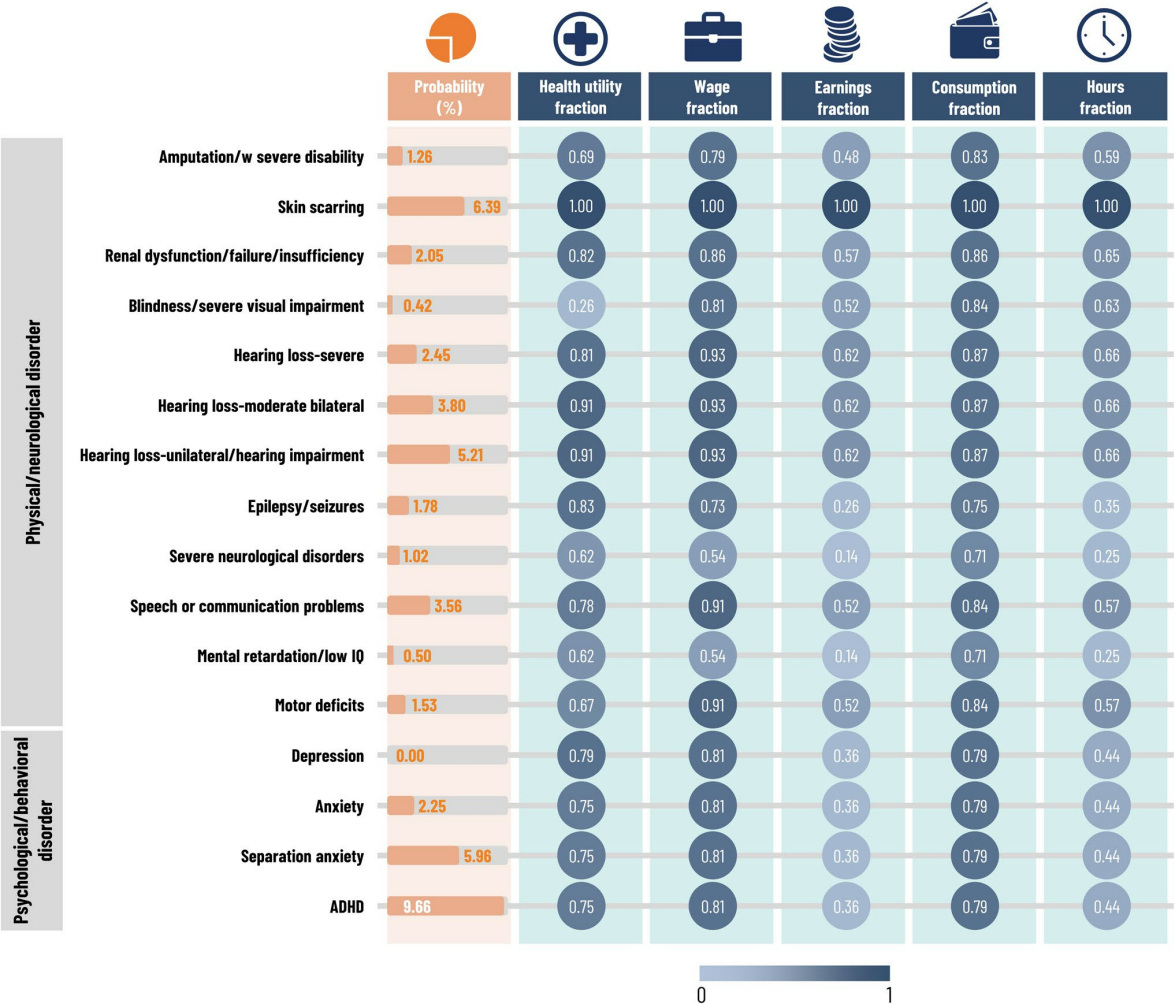
Lifecycle model inputs for sequelae, probability of sequelae, and respective fraction impact on health utility, wages, earnings, consumption, and hours considered. *ADHD* attention deficit hyperactivity disorder, *IQ* intelligent quotient, *w/* with

Given historical variability in incidence, we used age-specific incidence rates averaged over 2000 to 2014 [38], a period prior to 4CMenB introduction. Age-specific CFRs were from Ladhani et al. [39] and Shigematsu et al. [40]. We took risks of non-MenB-related deaths from 2013 to 2015 National Life Tables [41] and MenB sequelae probabilities from disability prevalences among survivors (Fig. 2) [42–48]. Figure 2 shows health utilities associated with permanent disabilities. In the acute phase, health utility fell to 0.065 and recovered linearly over 50 days [49]. General population health utilities were from Kind et al. [50].

Vaccine efficacies were 0%, 80%, and 82.8% for doses 1 and 2 and the booster (Portuguese PT-BEST study [51]). Mean durations of protection were 33, 33, and 38 months [52, 53].

### HALM

#### Constrained utility maximization problem

We derived individual WTP for health gains from a HALM, a budget-constrained individual lifetime utility maximization model based on Murphy and Topel [54]. The individual maximizes the expected present discounted value (EPDV) of lifetime utility. Expectations are taken over age and Markov state combinations $(i,j)$. For each such combination, the individual takes as given its associated probability ${s}_{j}\left(i\right)$ (determined in part by vaccination status), health utility ${q}_{j}\left(i\right)$, and hourly wage ${w}_{j}\left(i\right)$ and decided on optimal consumption ${c}_{j}\left(i\right)$ and non-market time ${l}_{j}\left(i\right)$. Given a time endowment $T$, market time is $T-{l}_{j}\left(i\right)$, and earnings are ${y}_{j}\left(i\right)={w}_{j}\left(i\right)*(T-{l}_{j}\left(i\right))$. Expected utility in each $(i,j)$ combination (“state utility”) is ${s}_{j}\left(i\right)*{q}_{j}(i)*u\left(z({c}_{j}\left(i\right),{l}_{j}\left(i\right))\right)$, that is, a product of the state probability, health utility, and economic utility $u$ from composite commodity consumption $z$; $u$ has constant-relative-risk aversion (CRRA) form; and $z$ is a constant elasticity of substitution (CES) function of consumption and non-market time. Non-market time consists of unpaid work and leisure. The multiplicative structure of expected period utility implies that health and economic goods are natural complements: the value of health was higher when economic goods were higher and vice versa.

Specifying the budget constraint requires three assumptions regarding capital markets, specifically regarding credit markets (for borrowing and saving), annuities (for insuring consumption against longevity risk), and disability insurance (for insuring consumption against lifetime earnings shocks from permanent disability).

The simplest assumption is perfect capital markets (PCM), which assumed perfect credit, annuity, and disability insurance markets. Under PCM, the budget constraint reduces to the requirement that the EPDV across all $(i,j)$ of lifetime consumption does not exceed initial assets plus the EPDV across all $(i,j)$ of lifetime earnings. Solving the utility maximization problem assuming PCM yields optimal values for ${c}_{j}\left(i\right),{l}_{j}\left(i\right)$ that has some implausible aspects: consumption that seems too high in early adult life, late life, and in the disabled states, such implausibility resulting from the assumptions of perfect borrowing, annuitization, and disability insurance, respectively.

Reality appears closer to ICM. In particular, public and private disability insurance (including in the UK) is often imperfect [6, 55, 56]. In such cases, reduced earnings potential from permanent disability results in lost income and consumption. Thus, with ICM, preventing permanent disability has instrumental value by preventing the permanent disruption to productivity, earnings, and consumption that occur in the absence of disability insurance. We call this instrumental value the financial risk protection (FRP) benefit of vaccination. Furthermore, early-life inability to borrow against future income implies consumption rising with age in early adulthood, and annuitization difficulties implies consumption declining with age in late life, both implications we find plausible. Thus, ICM was our base case assumption regarding capital markets.

However, given the technical challenges of modeling an ICM budget within the HALM, we instead adopted a pragmatic two-stage approach to our ICM base case. First, we solved the maximization problem using the simpler PCM budget constraint, and, from this solution, derived expressions for the value of a statistical life year (VSLY) as functions of consumption ${c}_{j}\left(i\right)$, non-market time ${l}_{j}\left(i\right)$, and wages ${w}_{j}\left(i\right)$. Second, we eschewed the ${c}_{j}\left(i\right),{l}_{j}\left(i\right)$ values implied by PCM and instead populated the VSLY expressions with values of consumption, non-market time, and wages that we estimated would be obtained under ICM. As discussed below, the intuition behind the VSLY expressions carries over from the PCM case to the ICM case so our pragmatic approach used both plausible formulas and inputs into those formulas.

Solving the maximization problem assuming PCM gives optimal ${c}_{j}\left(i\right),{l}_{j}(i)$ for all $(i,j)$. Given such solution and for each $(i,j)$ combination, we derived the individual’s WTP for an increase in the probability ${s}_{j}\left(i\right)$ of being in that state, which we call the VSLY and is given by:

1 $$\begin{array}{*{20}c} {{\text{VSLY}}_{j} \left( i \right) = \frac{{q_{j} \left( i \right)*u\left( {z\left( {c_{j} \left( i \right),l_{j} \left( i \right)} \right)} \right)}}{{q_{j} \left( i \right)*u_{c} \left( {c_{j} \left( i \right),l_{j} \left( i \right)} \right)}} + \left( {y_{j} \left( i \right) - c_{j} \left( i \right)} \right).} \\ \end{array}$$

${\text{VSLY}}$ is a function of the intrinsic and instrumental value of an increase in the probability of being in each state. Intrinsic value represents the goodness of that increased probability in and of itself, independently of its causal impact on consumption and non-market time. Such intrinsic value (the ratio in $(1)$) is the level of utility in a state (${q}_{j}*u$), monetized by dividing with the marginal utility of consumption (${q}_{j}*{u}_{c}$) in that state. Instrumental value has to do with the increased probability’s impact on lifetime consumption and non-market time, mediated by its impact on the budget constraint. Such impact equals net savings (the difference between income (${y}_{j}$) and consumption $({c}_{j})$), representing how much consumption in other states and ages is facilitated by being in that state. The expressions for intrinsic and instrumental values are economically plausible even when markets were imperfect: a state’s intrinsic value should center on the utility of that state, and net savings is an approximate measure of how much being in that state instrumentally facilitated consumption at other states and times.

The VSLY formula clarifies that:

- Health’s value varies by age and health state along with consumption, non-market time, and net savings, which implies that the CUA equal-value-per-QALY assumption fails to hold in more general lifecycle models like ours.
- Health’s value reflects not only earnings as in the standard human capital approach (where it proxies for consumption), but also unpaid work and leisure.

#### Calibrating consumption and non-market time

${VSLY}_{j}(i)$ Is a function of age- and state-specific consumption and non-market time values ${c}_{j}\left(i\right), {l}_{j}\left(i\right)$. We estimated what these values would have been under ICM for the following situations and plug these into (1) to derive ${VSLY}_{j}(i)$ corresponding to such situations.

##### Children

We assumed children younger than 16 did no paid work and devoted all their time to non-market activities. We assumed parents ensured children’s consumption was unaffected by disability, so consumption in all health states equaled that in the uninfected state, which in turn equaled that of children in the general population [55, 56]. We refer to the derived values for children as ${VSLY}_{j}^{c}(i)$ for $i=0,\dots ,15$.

##### Adults

For the uninfected state $(j=2)$, we used consumption, time use, and wage data from the UK general population [57] to estimate age-specific full consumption (equal to the sum of consumption and the value of non-market time, where the value of non-market time equaled the product of the wage and hours of non-market time). We then applied HALM optimality conditions to determine optimal consumption and non-market time conditional on full consumption (note that the resulting optimal consumption and non-market time are different from the data on consumption and non-market time used to estimate full consumption). For disabled states, we used evidence from the UK and (where unavailable) US (further details given in Supplementary Appendix 1) [58] to estimate the impact of disability on wages, consumption, and non-market time. We refer to the derived values as ${VSLY}_{j}^{icm}(i)$ for $i>15$.

Given the ${VSLY}_{j}^{c}(i)$ values for children and ${VSLY}_{j}^{icm}(i)$ values for adults, the individual’s or private WTP (PWTP) for vaccination is the present discounted value (PDV) across all age-state combinations of the vaccine-induced change in the probability of that combination multiplied by the VSLY of that combination (which for each state $j$ equals ${VSLY}_{j}^{c}(i)$ for $i\le 15$ and ${VSLY}_{j}^{icm}(i)$ for $i>15$). We denote this as private WTP by ${PWTP}^{icm}$.

##### PCM

We also computed PWTP under PCM and denoted this by ${PWTP}^{pcm}$. For the uninfected state, we set full consumption, consumption, and non-market time as we did for the ICM case. In contrast, for the disabled states, we used HALM optimality conditions (which presume perfect disability insurance) to determine consumption and non-market time. These yielded ${VSLY}_{j}^{pcm}(i)$ for $i>15$. We assumed ${VSLY}_{j}^{pcm}\left(i\right)={VSLY}_{j}^{c}(i)$ for children, and ${PWTP}^{pcm}$ follows.

We took the difference ${PWTP}^{icm}-{PWTP}^{pcm}$ to equal the FRP value of vaccination and denote this value ${PWTP}^{frp}$. We also refer to ${PWTP}^{pcm}$ as the residual (i.e., over-and-above FRP) intrinsic and instrumental value of vaccination.

#### Parental spillovers

To capture parental spillover benefits (PSB), we assumed that for each individual with MenB-related permanent disability, one parent faced an annual increased risk of depression of 0.17 (based on Al-Janabi et al. [59]) for the rest of the parent’s life (assuming an average age of 32 years) [60], which we valued using the depressed state components of ${VSLY}_{j}^{icm}$ to derive ${PWTP}^{psb}$.

#### Acute phase

We valued acute phase health utility loss using a value of a statistical disability (VSD) derived in Supplementary Appendix 1. Based on average hospital length of stay of 10 days [25], we also assumed one parent lost 7.14 days of earnings. We denote PWTP to avoid acute phase disability (APD) by ${PWTP}^{apd}$.

#### Other parameters

We obtained age-specific consumption and earnings from the National Transfer Accounts Project [61], time use from the UK Harmonized European Time Use Survey, and hourly wages from the UK Annual Survey of Hours and Earnings. The CRRA utility function requires an estimate of subsistence consumption, which we took to be half the absolute poverty rate from Allen et al. [62]. It also requires an estimate of the elasticity of intertemporal substitution, which we determined by setting the value of a statistical life (VSL) of a 41-year-old individual in the general population to be equal to £3.36 M, based on the upper range for VSL given by the Organisation for Economic Co-operation and Development (OECD) [63].

We obtained impacts of disability on hourly wages from Longhi et al. [64], and on hours worked from the Work Health and Disability Green Paper [65]. These jointly determined the impact of disability on earnings. In the absence of UK data, we relied on US evidence from Meyer and Mok [58] and assumed that the impact of disability on consumption was a third of its impact on earnings. Figure 2 summarizes the impact of disability on wages, earnings, consumption, and hours worked.

### Currency and costs

All currency units were in 2018 Great British Pounds (GBP) (this choice of year avoided non-representative COVID-induced price dynamics). Vaccination costs were £84.80 (including £9.80 administration cost) per dose [34] or $\pounds 254.40$ for three doses. Consistent with a previous 4CMenB CUA by Hammitt [21], we also considered costs relating to acute-stage medical treatment, long-term medical treatment, formal long-term caregiving, outbreak management, litigation, and special education, presented in Table 1.

**Table 1:** HALM parameter inputs for base case and scenarios considered

Parameter	Base case	Scenario A	Scenario B	Scenario C	Scenario D	Scenario F	Scenario G
		Change in VSL to £2.68 M	Addition of non-traditional elements consistent with Beck et al. [34]	Change in incidence base year to 2014	Higher vaccine efficacy	Consumption gap equals earnings gap	Lower health- related discount rates to 1.5%
Non-cost parameters							
Incidence of MenB [38]	Mean rate reported in England (2000–2014)	–	–	2014 incidence	–	–	–
Case fatality rate (%) [39, 40]	2.7–9.7%	–	–	–	–	–	–
Subsidence consumption	50% of basic needs at poverty line	–	–	–	–	–	–
Life cycle/capita consumption [61]	National Transfer Accounts project	–	–	–	–	–	–
Hourly earnings [57]	UK Annual Survey of Hours of Earnings						
Productivity loss: acute phase (days) [25]	7.14						
Vaccine efficacy (%) [51]		–	–	–		–	–
Dose 1	0.00				58.20		
Dose 2	80.00				83.20		
Dose 3	82.80				87.10		
Duration of protection (months) [52, 53]		–	–	–	–	–	–
Dose 1	33.00						
Dose 2	33.00						
Dose 3	38.00						
Discounting (%) [34]	3.50 (costs)	–	–	–	–	–	–
	3.50 (effects)						1.50 (effects)
Parental spillover: risk of depression for each MenB-related disability over parents’ lifetime [59]	0.17	–	–	–	–	–	–
QAF [34]	3	–	–	–	–	–	–
Cost parameters (2018, £)							
VSL [63]	3.36 M (2016)	2.68 M (2018)	–	–	–	–	–
Vaccine cost (all 3 doses) [34]	254.40 (84.80 per dose including administrative cost of 9.80)	–	–	–	–	–	–
Averted acute phase treatment costs (assumption based on Beck et al. (2021)) [34]	3.33	–	–	–	–	–	–
Averted long-term treatment costs (assumption based on Beck et al. (2021)) [34]	6.36	–	–	–	–	–	–
Averted long-term formal care costs (assumption based on Beck et al. (2021)) [34]	0.00	–	0.47	–	–	–	–
Averted special education costs (assumption based on Beck et al. (2021)) [34]	0.00	–	6.3	–	–	–	–
Averted litigation costs (assumption based on Beck et al. (2021)) [34]	0.00	–	0.36	–	–	–	–
Averted outbreak management costs (assumption based on Beck et al. (2021)) [34]	0.00	–	0.004	–	–	–	–

*HALM* health-augmented lifecycle model, *MenB* meningococcal serogroup B, *QAF* QALY adjustment factor, *QALY* quality-adjusted life year, *VSL* value of a statistical life. See the Supplementary Appendix for a full set of input data tables

### Scenario analysis

We performed the following one-way scenario analyses (see Table 1):

- Scenario A reduced VSL to a conservative £2.68 M, as proposed for EU-27 countries by an OECD meta-analysis [63].
- Scenario B introduced non-traditional cost elements used in Beck et al. [34]: formal long-term caregiving, outbreak management, litigation, and special education.
- Scenario C applied more conservative 2014 incidence rates from the European Centre for Disease Prevention and Control (ECDC) [66].
- Scenario D applied higher vaccine efficacy of 58.2%, 83.2%, and 87.1% following each dose [51].
- Scenario E allowed larger effects of disability on consumption in the absence of disability insurance, assuming a consumption gap the size of the earnings gap.
- Scenario F reduced the discount rate for health to 1.5% and retained the discount rate for costs at 3.5% [34].

### Overall private and social willingness to pay and rates of return

The overall PWTP and lifetime private rate of return are:

2 $$\begin{array}{*{20}c} {{\text{PWTP}} = {\text{PWTP}}^{frp} + {\text{PWTP}}^{pcm} + {\text{PWTP}}^{psb} + {\text{PWTP}}^{apd} .} \\ \end{array}$$

3 $$\begin{array}{*{20}c} {{\text{RoR}}^{p} = 100 \times \left( {\frac{{{\text{PWTP}}}}{vc - atc} - 1} \right),} \\ \end{array}$$

where $vc$ represents vaccination costs and $atc$ represents averted treatment costs, comprising averted acute phase treatment costs (AATC) and averted long-term treatment costs (ALTCC).

**Fig. 3:**
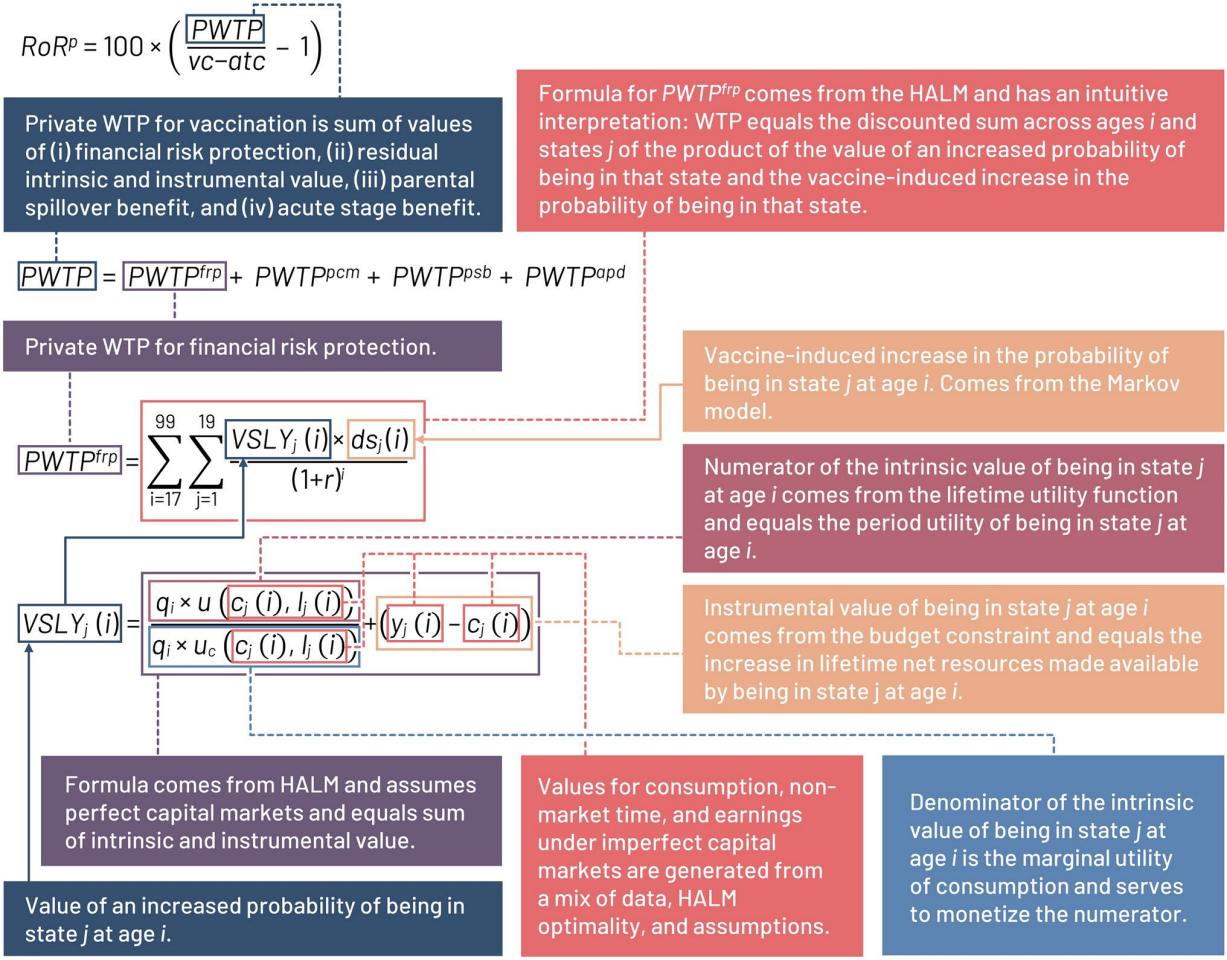
Visual representation of important determinants of the private rate of return. *apd* acute phase disability, *atc* averted treatment costs, *c* consumption, *ds* impact on vaccination, *frp* financial risk protection, *HALM* health-augmented lifecycle model, *i* any given year of life, *j* health state, *l* labor trade off, *pcm* perfect capital market, *psb* parental spillover benefits, *PWTP* private willingness to pay, *q* health utility, *r* rate, *RoR* rate of return, u period utility, *WTP* willingness to pay, *v* vaccination, *vc* vaccination cost, *VSLY* value of a statistical life year, *y* earnings

Figure 3 visually represents the determination of ${RoR}^{p}$ and one of its central elements, FRP, by the various parts of our modeling approach.

We follow JCVI and Beck et al. in assuming that society attributes extra value to preventing severe disease (or that there are “social severity preferences”) and that such value can be modeled by scaling up long-term costs of MenB (or equivalently, long-term gains from 4CMenB) by 3. [14, 25, 34] We apply this scale factor of 3 to the long-term benefits to the vaccinated infant, which includes the financial risk protection value ${PWTP}^{frp}$ and the residual intrinsic and instrumental value ${PWTP}^{pcm}$, but excludes parental spillovers ${PWTP}^{psb}$ and acute phase burdens ${PWTP}^{apd}$. This yields a social WTP (SWTP) and lifetime social rate of return of:

4 $$\begin{array}{*{20}c} {{\text{SWTP}} = 3 \times {\text{PWTP}}^{frp} + 3 \times {\text{PWTP}}^{pcm} + {\text{PWTP}}^{psb} + {\text{PWTP}}^{apd} .} \\ \end{array}$$

5 $$\begin{array}{*{20}c} {{\text{RoR}}^{s} = 100 \times \left( {\frac{{{\text{SWTP}}}}{vc - atc} - 1} \right).} \\ \end{array}$$

We revised our RoR formulas for scenario B to include other novel cost elements: averted litigation costs (ALC), averted outbreak management costs (AOC), averted long-term formal care costs (ALFCC) and averted special education costs (ASEC):

6 $$\begin{array}{*{20}c} {{\text{RoR}}_{B}^{p} = 100 \times \left( {\frac{{{\text{PWTP}} + alfcc + a\sec }}{vc - atc - alc - aoc} - 1} \right),} \\ \end{array}$$

and

7 $$\begin{array}{*{20}c} {{\text{RoR}}_{B}^{s} = 100 \times \left( {\frac{{{\text{SWTP}} + alfcc + a\sec }}{vc - atc - alc - aoc} - 1} \right).} \\ \end{array}$$

Note that averted costs within the health payer’s budget were cost offsets in the denominator, while other averted costs were benefits in the numerator. This allowed us to interpret RoR as returns per GBP paid out of the health payer’s budget.

## Results

**Fig. 4:**
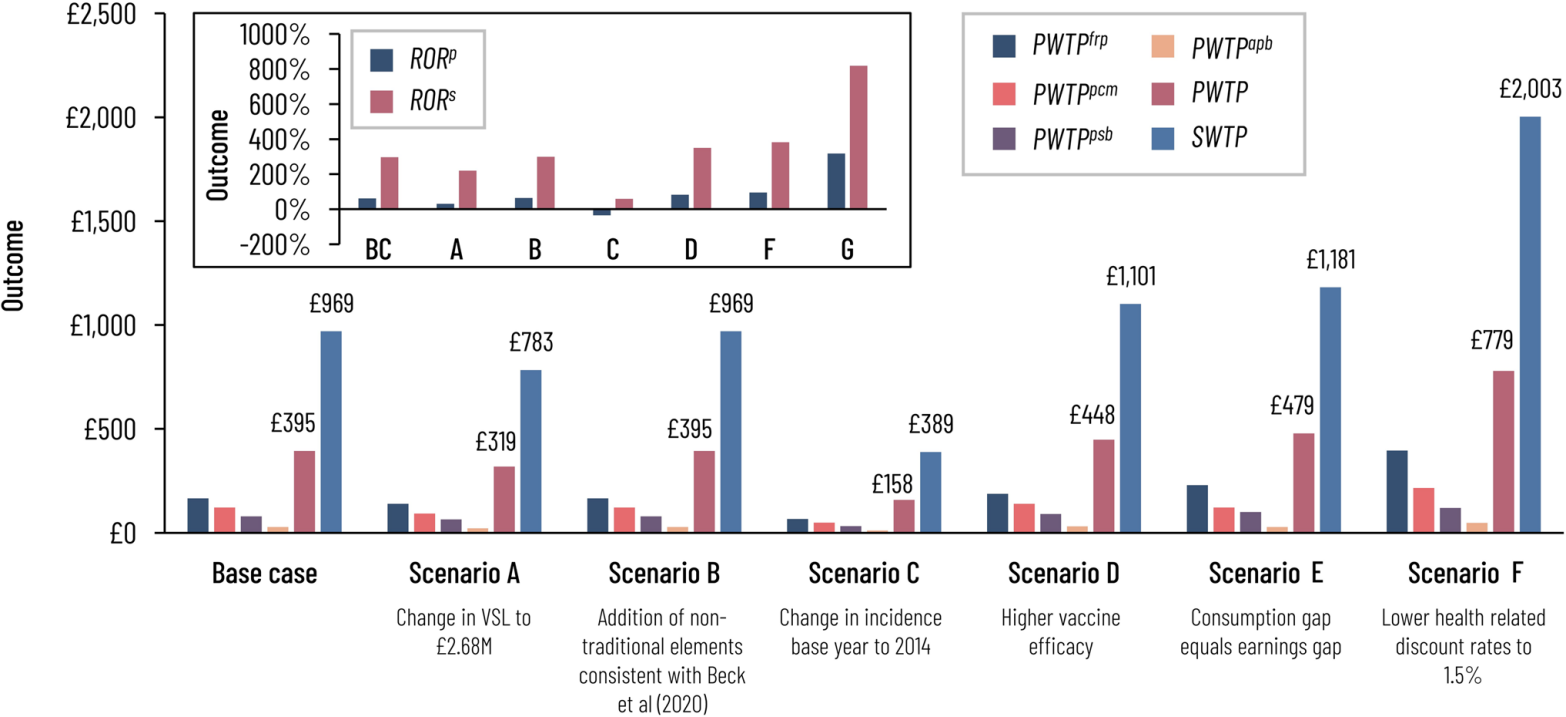
Base case and scenario model outcomes. *apd* acute phase disability, *BC* base case, *frp* financial risk protection, *M* million, *p* private, *pcm* perfect capital market, *psb* parental spillover benefits, *PWTP* private willingness to pay, r rate, *RoR* rate of return, *s* social, *SWTP* social willingness to pay, *VSL* value of statistical life, *WTP* willingness to pay

Figure 4 summarizes base case and scenario analysis results.

Vaccination produced a health gain (excluding parental spillovers) of 0.43 quality-adjusted days of life per infant. ${\text{PWTP}}$ for vaccination was £394.51, consisting of £165.54 in FRP value, £79.31 in PSB, £27.71 in APD value, and £121.95 in residual intrinsic and instrumental value of health. The ${\text{SWTP}}$ was £969.49. Averted costs per vaccinated infant included £3.33 in acute treatment costs and £6.36 in long-term treatment costs. The baseline lifetime private and social RoRs were 61% and 296%, respectively.

In scenario analyses, reducing the VSL (Scenario A) lowered the private and social RoRs to 30% and 220%, respectively. Including non-traditional cost categories in Scenario B raised private and social RoRs to 64% and 300%, respectively. Replacing the average incidence with more recent incidence (Scenario C) reduced private and social RoRs to − 35% and 59%, respectively. Raising vaccine efficacy (Scenario D) raised the private and social RoRs to 83% and 350%, respectively. Setting the consumption gap equal to the earnings gap (Scenario E) raised the private and social RoRs to 96% and 383%, respectively. Applying differential discounting (Scenario F) raised the private and social RoRs to 318% and 819%, respectively.

## Discussion

### Policy implications of our results

The estimated RoRs inform decisions by the UK Parliament, Government, and DHSC. The HALM allows individuals to either consume or save at the market interest rate, implying that the RoRs internalize individuals’ opportunity cost in terms of foregone consumption or savings. The 61% private RoR in the face of foregone consumption and savings suggests it is good VfM for the UK Parliament to finance the vaccine through taxation. Absent a familiar UK benchmark RoR, we consider public spending on female schooling in low- to middle-income countries, generally considered a global best buy. Based on a previous study, we estimate the RoR to such spending, incorporating both earnings and longevity benefits, at 280% [67]. That study had shorter time horizon than ours, extending only till retirement, in contrast to our 100-year horizon. Subject to that caveat (a caveat mitigated by discounting), in the base case, our private RoR did not meet this 280% threshold, but the social RoR did, suggesting that based on the social RoR, infant MenB vaccination could be attractive relative to that of non-health public expenditure and would therefore be worth accommodating through an expansion in the DHSC budget.

Claxton et al. estimated the ICER of the marginal health technology in the UK at approximately £15,000 per QALY [68]. The UK Green Book provided a WTP per QALY of £60,000 [16]. These figures suggest an RoR to the marginal health technology of ((60,000/15,000)-1)*100 = 300%. The private RoR obtained falls below this value, but our social RoR is very close, which suggests that based on the social RoR, infant MenB vaccination could be good VfM for the DHSC, given its fixed budget. These findings are consistent with those of Beck et al. (2021) and with the DHSC’s decision to fund infant 4CMenB [34].

We conclude that, based on private values across our base case and scenario analyses, the UK Parliament would be justified in raising taxes to expand UK Government and DHSC budgets to accommodate infant 4CMenB. However, the UK Government and the DHSC would not be justified in funding it out of existing budgets (echoing conventional CUA conclusions) [34]. However, based on social values, infant 4CMenB is good VfM for all three decision-makers, except when VSL and incidence rates are lower than in the base case. The most policy-consequential factors we identify are social severity preferences, baseline incidence, discounting, VSL, FRP, and the residual instrumental and intrinsic values of health. Considerably less important are PSB, consumption sensitivity to earnings decline, APD, vaccine effectiveness, averted treatment costs, and novel cost elements.

### Limitations and future work

The HALM is complex, but such complexity, we believe, is particularly justified in applications where there are important but complex interactions between health, on the one hand, and microeconomic quantities like earnings, consumption, and time use, on the other. Such is the case with MenB given its impact on long-term disability the microeconomic consequences of such disability.

An advisory board suggests the need for continued methodological development of the HALM, including deeper investigation of validity and robustness. One priority area is to validate our “pragmatic” approach to ICM (in which we derive value formulas from a maximization problem that assumes a PCM budget constraint, but in which we populate those formulas with inputs that we assume reflect the workings of ICM). Such validation can be done by developing a fully theoretically rigorous version of the HALM where the budget constraint reflects ICM and comparing our results to those of such a version. A second priority area is to validate the approach of representing social severity preferences by multiplying long-term costs and benefits by 3. Such validation can be done by deriving social severity weights in a fully rigorous way using SWFs and comparing the resulting weights to 3 [69]. Within the HALM, the natural complementarity between health and consumption can have potentially inequitable implications: the value of health would be higher for those with higher levels of consumption (e.g., the non-disabled). Linking the HALM to SWFs could also help redress those implications. A third priority area is investigation of empirical robustness: the evidence base we rely on for the long-term impact of disability on wages, earnings, consumption, and time use is underdeveloped and should be strengthened. Fourth and finally, we simplified by assuming no elevated mortality risk from permanent disability. Such assumption can be relaxed, and the results made more empirically robust, by relying on evidence on such elevated risk from references such as Shen et al. [37].

## Supplementary Information

Below is the link to the electronic supplementary material.

Supplementary file1 (DOCX 137 KB)

## Data Availability

All data used in the project are in the public domain. This research did not involve any human subjects.

## References

[1] World Health Organization (WHO). Meningitis - Serogroup distribution of invasive meningococcal disease, 2019. 2020; https://www.who.int/health-topics/meningitis#tab=tab_1. Cited 30 Jun 2022

[2] Booy, R., Gentile, A., Nissen, M., Whelan, J., Abitbol, V.: Recent changes in the epidemiology of Neisseria meningitidis serogroup W across the world, current vaccination policy choices and possible future strategies. Hum. Vaccin. Immunother. **15**(2), 470–480 (2019) 30296197 10.1080/21645515.2018.1532248 PMC6505668

[3] Beebeejaun, K., Parikh, S.R., Campbell, H., Gray, S., Borrow, R., Ramsay, M.E., Ladhani, S.N.: Invasive meningococcal disease: timing and cause of death in England, 2008–2015. J. Infect. **80**(3), 286–290 (2020) 31904388 10.1016/j.jinf.2019.12.008

[4] Olbrich, K.J., Müller, D., Schumacher, S., Beck, E., Meszaros, K., Koerber, F.: Systematic review of invasive meningococcal disease: sequelae and quality of life impact on patients and their caregivers. Infect. Dis. Ther. **7**(4), 421–438 (2018) 30267220 10.1007/s40121-018-0213-2 PMC6249177

[5] Scholz, S., Koerber, F., Meszaros, K., Fassbender, R.M., Ultsch, B., Welte, R., Greiner, W.: The cost-of-illness for invasive meningococcal disease caused by serogroup B Neisseria meningitidis (MenB) in Germany. Vaccine. **37**(12), 1692–1701 (2019) 30661834 10.1016/j.vaccine.2019.01.013

[6] Wright, C., Wordsworth, R., Glennie, L.: Counting the cost of meningococcal disease: scenarios of severe meningitis and septicemia. Paediatr. Drugs **15**(1), 49–58 (2013) 23322553 10.1007/s40272-012-0006-0

[7] Joint Committee on Vaccination and Immunisation. JCVI interim position statement on use of Bexsero® meningococcal B vaccine in the UK. 2013. https://assets.publishing.service.gov.uk/media/5a7c6caeed915d696ccfcb03/JCVI_interim_statement_on_meningococcal_B_vaccination_for_web.pdf. Cited 11 October 2023

[8] Petitions UK Goverment and Parliament. Give the Meningitis B vaccine to all children, not just newborn babies. 2016. https://petition.parliament.uk/archived/petitions/108072. Cited 13 Dec 2018

[9] Joint Committee on Vaccination and Immunisation. Code of Practice June 2013. 2013 https://assets.publishing.service.gov.uk/media/5a7b9b3440f0b62826a04a80/JCVI_Code_of_Practice_revision_2013_-_final.pdf. Cited 18 Oct 2023

[10] Joint Committee on Vaccination and Immunisation. JCVI position statement on use of Bexsero® meningococcal B vaccine in the UK. 2014. https://assets.publishing.service.gov.uk/government/uploads/system/uploads/attachment_data/file/294245/JCVI_Statement_on_MenB.pdf. Cited 18 Oct 2023

[11] Christensen, H., Hickman, M., Edmunds, W.J., Trotter, C.L.: Introducing vaccination against serogroup B meningococcal disease: an economic and mathematical modelling study of potential impact. Vaccine. **31**(23), 2638–2646 (2013) 23566946 10.1016/j.vaccine.2013.03.034 PMC3743045

[12] Raftery J. Should the NHS use the new meningitis B vaccine? BMJ Opinion. 28 (2014)

[13] Wise, J.: Meningitis B vaccine to be introduced in UK after U turn on its cost effectiveness. BMJ Brit Med J. **348**, g2327 (2014) 24665138 10.1136/bmj.g2327

[14] Joint Committee on Vaccination and Immunisation. Minute of the meeting on Tuesday 11 and Wednesday 12 February 2014. 2014 Feb 12. https://app.box.com/s/iddfb4ppwkmtjusir2tc/file/229171703722. Cited 18 Oct 2023

[15] Ladhani, S.N., Campbell, H., Parikh, S.R., Saliba, V., Borrow, R., Ramsay, M.: The introduction of the meningococcal B (MenB) vaccine (Bexsero®) into the national infant immunisation programme–new challenges for public health. J. Infect. **71**(6), 611–614 (2015) 26433141 10.1016/j.jinf.2015.09.035

[16] HM Treasury. The green book: central government guidance on appraisal and evaluation. Crown pp 138 (2022)

[17] Turner, H.C., Archer, R.A., Downey, L.E., Isaranuwatchai, W., Chalkidou, K., Jit, M., Teerawattananon, Y.: An introduction to the main types of economic evaluations used for informing priority setting and resource allocation in healthcare: key features, uses, and limitations. Front. Public Health **9**, 722927 (2021) 34513790 10.3389/fpubh.2021.722927 PMC8424074

[18] Robinson, L.A., Hammitt, J.K., Chang, A.Y., Resch, S.: Understanding and improving the one and three times GDP per capita cost-effectiveness thresholds. Health Policy Plan. **32**(1), 141–145 (2017) 27452949 10.1093/heapol/czw096

[19] World Health Organization (WHO). Evidence, policy, impact: WHO guide for evidence-informed decision-making. 2022. https://www.who.int/publications/i/item/9789240039872. Cited 27 Jan 2022

[20] Bleichrodt, H., Quiggin, J.: Life-cycle preferences over consumption and health: when is cost-effectiveness analysis equivalent to cost–benefit analysis? J. Health Econ. **18**(6), 681–708 (1999) 10847930 10.1016/s0167-6296(99)00014-4

[21] Hammitt, J.K.: Admissible utility functions for health, longevity, and wealth: integrating monetary and life-year measures. J. Risk Uncertain. **47**(3), 311–325 (2013)

[22] Bertram, M.Y., Lauer, J.A., De Joncheere, K., Edejer, T., Hutubessy, R., Kieny, M.P., Hill, S.R.: Cost-effectiveness thresholds: pros and cons. Bull. World Health Organ. **94**(12), 925–930 (2016) 27994285 10.2471/BLT.15.164418 PMC5153921

[23] Adler, M.: A better calculus for regulators: from cost–benefit analysis to the social welfare function. Duke Law School Public Law & Legal Theory Series. pp 2017–19 (2017)

[24] Christensen, H., Irving, T., Koch, J., Trotter, C.L., Ultsch, B., Weidemann, F., et al.: Epidemiological impact and cost-effectiveness of universal vaccination with Bexsero((R)) to reduce meningococcal group B disease in Germany. Vaccine. **34**(29), 3412–3419 (2016) 27109566 10.1016/j.vaccine.2016.04.004

[25] Christensen, H., Trotter, C.L., Hickman, M., Edmunds, W.J.: Re-evaluating cost effectiveness of universal meningitis vaccination (Bexsero) in England: modelling study. BMJ (Clin. Res. Ed). **9**(349), g5725 (2014) 10.1136/bmj.g5725 PMC4192138 25301037

[26] Gasparini, R., Landa, P., Amicizia, D., Icardi, G., Ricciardi, W., de Waure, C., et al.: Vaccinating Italian infants with a new multicomponent vaccine (Bexsero(R)) against meningococcal B disease: a cost-effectiveness analysis. Hum. Vaccin. Immunother. **12**(8), 2148–2161 (2016) 27163398 10.1080/21645515.2016.1160177 PMC4994748

[27] Ginsberg, G.M., Block, C., Stein-Zamir, C.: Cost–utility analysis of a nationwide vaccination programme against serogroup B meningococcal disease in Israel. Int. J. Public Health **61**(6), 683–692 (2016) 27105884 10.1007/s00038-016-0821-0

[28] Hanquet G, Christensen H, Agnew E, Trotter C, Robays J, Dubois C, et al. A quadrivalent vaccine against serogroup B meningococcal disease: a cost-effectiveness study. 2014; https://kce.fgov.be/en/publications/all-reports/a-quadrivalent-vaccine-against-serogroup-b-meningococcal-disease-a-cost-effectiveness-study. 6 Dec 2022

[29] Lecocq, H., Parent-du-Chatelet, I., Taha, M.K., Levy-Bruhl, D., Dervaux, B.: Epidemiological impact and cost-effectiveness of introducing vaccination against serogroup B meningococcal disease in France. Vaccine. **34**(19), 2240–2250 (2016) 27002504 10.1016/j.vaccine.2016.03.020

[30] Pouwels, K.B., Hak, E., van der Ende, A., Christensen, H., van den Dobbelsteen, G.P., Postma, M.J.: Cost-effectiveness of vaccination against meningococcal B among Dutch infants: crucial impact of changes in incidence. Hum. Vaccin. Immunother. **9**(5), 1129–1138 (2013) 23406816 10.4161/hv.23888 PMC3899149

[31] Tirani, M., Meregaglia, M., Melegaro, A.: Health and economic outcomes of introducing the new MenB vaccine (Bexsero) into the Italian routine infant immunisation programme. PLoS ONE **10**(4), e0123383 (2015) 25874805 10.1371/journal.pone.0123383 PMC4395261

[32] Tu, H.A., Deeks, S.L., Morris, S.K., Strifler, L., Crowcroft, N., Jamieson, F.B., et al.: Economic evaluation of meningococcal serogroup B childhood vaccination in Ontario Canada. Vaccine. **32**(42), 5436–5446 (2014) 25131732 10.1016/j.vaccine.2014.07.096

[33] Bonanni, P., Villani, A., Scotti, S., Biasci, P., Russo, R., Maio, T., et al.: The recommended lifetime immunization schedule from the board of vaccination calendar for life in Italy: a continuing example of impact on public health policies. Vaccine. **39**(8), 1183–1186 (2021) 33589048 10.1016/j.vaccine.2021.01.019

[34] Beck, E., Klint, J., Neine, M., Garcia, S., Meszaros, K.: Cost-effectiveness of 4CMenB infant vaccination in England: a comprehensive valuation considering the broad impact of Serogroup B invasive meningococcal disease. Value Health. **24**(1), 91–104 (2021) 33431159 10.1016/j.jval.2020.09.004

[35] Bom, P.R., Ligthard, J.E.: What have we learned from three decades of research on the productivity of public capital? J. Econ. Surv. **28**(5), 889–916 (2013)

[36] Shen, J., Begum, N., Ruiz-Garcia, Y., Martinon-Torres, F., Bekkat-Berkani, R., Meszaros, K.: Range of invasive meningococcal disease sequelae and health economic application—a systematic and clinical review. BMC Public Health **22**(1), 1078 (2022) 35641955 10.1186/s12889-022-13342-2 PMC9153861

[37] Shen, J., Bouee, S., Aris, E., Emery, C., Beck, E.C.: Long-term mortality and state financial support in invasive meningococcal disease-real-world data analysis using the French National Claims Database (SNIIRAM). Infect. Dis. Ther. **11**(1), 249–262 (2022) 34791633 10.1007/s40121-021-00546-z PMC8847620

[38] Public Health England. Invasive meningococcal infections laboratory reports in England by capsular group, age group & calendar year, 2000–2014. p. 2 (2015)

[39] Ladhani, S.N., Giuliani, M.M., Biolchi, A., Pizza, M., Beebeejaun, K., Lucidarme, J., et al.: Effectiveness of meningococcal B vaccine against endemic hypervirulent neisseria meningitidis W Strain England. Emerg. Infect. Dis. **22**(2), 309–311 (2016) 26811872 10.3201/eid2202.150369 PMC4734511

[40] Shigematsu, M., Davison, K.L., Charlett, A., Crowcroft, N.S.: National enhanced surveillance of meningococcal disease in England, Wales and Northern Ireland, January 1999–June 2001. Epidemiol. Infect. **129**(3), 459–470 (2002) 12558328 10.1017/s0950268802007549 PMC2869907

[41] Office for National Statistics (ONS). National Life Tables, Great Britain, 1980–1982 to 2013–2015. https://www.ons.gov.uk/file?uri=/peoplepopulationandcommunity/birthsdeathsandmarriages/lifeexpectancies/datasets/nationallifetablesgreatbritainreferencetables/current/nltgb1315reg.xls. Cited 5 Apr 2020

[42] Bennett, J.E., Sumner, W., 2nd., Downs, S.M., Jaffe, D.M.: Parents’ utilities for outcomes of occult bacteremia. Arch. Pediatr. Adolesc. Med. **154**(1), 43–48 (2000) 10632249

[43] Bettinger, J.A., Scheifele, D.W., Le Saux, N., Halperin, S.A., Vaudry, W., Tsang, R., Members of Canadian Immunization Monitoring Program: The disease burden of invasive meningococcal serogroup B disease in Canada. Pediatr. Infect. Dis. J. **32**(1), e20–e25 (2013) 22926207 10.1097/INF.0b013e3182706b89

[44] Blakeney, P., Meyer, W., 3rd., Robert, R., Desai, M., Wolf, S., Herndon, D.: Long-term psychosocial adaptation of children who survive burns involving 80% or greater total body surface area. J. Trauma **44**(4), 625–632 (1998). (**discussion 33–4**) 9555833 10.1097/00005373-199804000-00011

[45] Brown, M.M., Brown, G.C., Sharma, S., Kistler, J., Brown, H.: Utility values associated with blindness in an adult population. Br. J. Ophthalmol. **85**(3), 327–331 (2001) 11222340 10.1136/bjo.85.3.327 PMC1723892

[46] Saarni, S.I., Suvisaari, J., Sintonen, H., Pirkola, S., Koskinen, S., Aromaa, A., Lönnqvist, J.: Impact of psychiatric disorders on health-related quality of life: general population survey. Brit. J. Psychiatry. **190**, 326–332 (2007) 17401039 10.1192/bjp.bp.106.025106

[47] Viner, R.M., Booy, R., Johnson, H., Edmunds, W.J., Hudson, L., Bedford, H., et al.: Outcomes of invasive meningococcal serogroup B disease in children and adolescents (MOSAIC): a case-control study. Lancet Neurol. **11**(9), 774–783 (2012) 22863608 10.1016/S1474-4422(12)70180-1

[48] Wyld, M., Morton, R.L., Hayen, A., Howard, K., Webster, A.C.: A systematic review and meta-analysis of utility-based quality of life in chronic kidney disease treatments. PLoS Med. **9**(9), e1001307 (2012) 22984353 10.1371/journal.pmed.1001307 PMC3439392

[49] Kennedy, I.T.R., van Hoek, A.J., Ribeiro, S., Christensen, H., Edmunds, W.J., Ramsay, M.E., Ladhani, S.N.: Short-term changes in the health state of children with group B meningococcal disease: A prospective, national cohort study. PLoS ONE **12**(5), e0177082 (2017) 28545152 10.1371/journal.pone.0177082 PMC5436659

[50] Kind, P., Hardman, G., Macran, S.: UK population norms for EQ-5D, p. 98. The University of York—Centre for Health Economics, York (1999)

[51] Rodrigues, F.: Portuguese Meningococcus Group B Vaccine Effectiveness Study (PT-BEST)—preliminary results. In: 15th European Meningococcal and Haemophilus Disease Society (EMGM) Congress; Lisbon, Portugal (2019)

[52] Martinon-Torres, F., Carmona Martinez, A., Simko, R., Infante Marquez, P., Arimany, J.L., Gimenez-Sanchez, F., et al.: Antibody persistence and booster responses 24–36 months after different 4CMenB vaccination schedules in infants and children: a randomised trial. J. Infect. **76**(3), 258–269 (2018) 29253560 10.1016/j.jinf.2017.12.005

[53] Martinon-Torres, F., Safadi, M.A.P., Martinez, A.C., Marquez, P.I., Torres, J.C.T., Weckx, L.Y., et al.: Reduced schedules of 4CMenB vaccine in infants and catch-up series in children: immunogenicity and safety results from a randomised open-label phase 3b trial. Vaccine. **35**(28), 3548–3557 (2017) 28533054 10.1016/j.vaccine.2017.05.023

[54] Murphy, K.M., Topel, R.H.: The value of health and longevity, p. 96. National Bureau of Economic Research, Cambridge (2005)

[55] Morciano, M., Hancock, R., Pudney, S.: Disability costs and equivalence scales in the older population in Great Britain. Rev. Income Wealth. **61**(3), 494–514 (2014)

[56] Zaidi, A., Burchardt, T.: Comparing incomes when needs differ: equivalization for the extra costs of disability in the UK. Rev. Income Wealth. **51**(1), 89–114 (2005)

[57] UK from the Annual Survey of Hours and Earnings. Median hourly earnings excluding overtime, provisional 2018 estimates.

[58] Meyer, B.D., Mok, W.K.C.: Disability, earnings, income and consumption. J. Public Econ. **171**, 51–69 (2019)

[59] Al-Janabi, H., Van Exel, J., Brouwer, W., Trotter, C., Glennie, L., Hannigan, L., Coast, J.: Measuring health spillovers for economic evaluation: a case study in meningitis. Health Econ. **25**(12), 1529–1544 (2016) 26464311 10.1002/hec.3259 PMC5111598

[60] Office for National Statistics (ONS). Births by parents’ characteristics in England and Wales: (2015)

[61] National Transfer Accounts Project. Labor Income and Private and public consumption other than health and education. Smooth Mean. United Kingdom 2012. 2019 http://www.ntaccounts.org/web/nta/show. Cited 5 April 2020

[62] Allen, R.C.: Absolute poverty: when necessity displaces desire. Am. Econ. Rev. **107**(12), 3690–3721 (2017)

[63] Organisation for Economic Co-operation and Development (OECD). Mortality risk valuation in environment, health, and transport policies. 2012 https://www.oecd.org/env/tools-evaluation/mortalityriskvaluationinenvironmenthealthandtransportpolicies.htm. Cited 4 May 2016

[64] Longhi, S.: The disability pay gap. Equality and Human Rights Commission; p. 95 (2017)

[65] UK Government—Department of Health. The work, health, and disability green paper: data pack. UK: Crown; (2016)

[66] European Centre for Disease Prevention and Control (ECDC). Surveillance Atlas of infectious diseases - Invasive Meningococcal Disease, Confirmed cases, Serogroup B notification rate, 2014, age-specific rate (Serogroup B cases)

[67] Pradhan E, Suzuki EM, Martinez S, Schaferhoff M, Jamison DT. The effects of education quantity and quality on child and adult mortality: their magnitude and their value. In: Bundy DAP, Silva ND, Horton S, Jamison DT, Patton GC, editors. Child and Adolescent Health and Development. 3rd ed. Washington (DC) (2017) 30212146

[68] Claxton, K., Martin, S., Soares, M., Rice, N., Spackman, E., Hinde, S., et al.: Methods for the estimation of the National Institute for Health and Care Excellence cost-effectiveness threshold. Health Technol. Assess. **19**(14), 1–503 (2015). (**v–vi**) 25692211 10.3310/hta19140 PMC4781395

[69] Fleurbaey, M., Luchini, S., Muller, C., Schokkaert, E.: Equivalent income and fair evaluation of health care. Health Econ. **22**(6), 711–729 (2013) 22767541 10.1002/hec.2859

