# Supplementary material for: Lifecycle model-based evaluation of infant 4CMenB vaccination in the UK
Source: Eur J Health Econ. 2024 Jan 5;25(7):1133–46. doi: 10.1007/s10198-023-01654-y (PMC11377635; doi:10.1007/s10198-023-01654-y)
Supplement: Supplementary file 1 — Supplementary file1 (DOCX 137 KB) [file 10198_2023_1654_MOESM1_ESM.docx]

**Supplementary Appendix 1**

Model assumptions, methods, inputs and calibration

**Supplement to:** Lifecycle model-based evaluation of infant 4CMenB vaccination in the UK

**1. Disease model**

***1.1. Notation***

We denote age in any given year of life by $i$ (where $0\leq i\leq100$) and health state by $j$ (where $j=1,\ldots,19$). Our Markov model yields an age-dependent period Markov transition matrix $\varphi(i)$ with representative element (where $r,c=1,\ldots,19$ denote row and column, respectively) (this is a left stochastic matrix with columns summing to one):

$$\begin{aligned} \varphi_{r,c}\left( i \right)=Prob\left( j=r at age i+1 | j=c at age i \right).\#\left( 1 \right) \end{aligned}$$

This representative element gives the probability of moving from one state to another, from one age to the next. From this, we can calculate the cumulative Markov transition matrix:

$$\begin{aligned} \Phi\left( i \right)=\prod_{l=a}^{i} \varphi\left( l \right).\#\left( 2 \right) \end{aligned}$$

In this cumulative matrix, the representative element is given by:

$$\begin{aligned} \Phi_{r,c}\left( i \right)=Prob\left( j=r at age i+1 | j=c at age 0 \right).\#\left( 3 \right) \end{aligned}$$

This representative element measures the probability of being in some state at some age (potentially long after model entry), conditional on being in some other state at model entry.

We denote the initial distribution of the cohort across the $j$ states by the $j$-dimensional column vector,

$$\begin{aligned} s\left( 0 \right)=\left[ 0,1,0,\ldots,0 \right],\#\left( 4 \right) \end{aligned}$$

whose entries reflect the shares of the cohort occupying each of the 19 states. We index states so that death is the first entry (i.e., death is $j=1$), the uninfected state is the second (i.e., uninfected is $j=2$), the temporarily disabled state is the third (i.e., temporary disability is $j=3$), and the remaining states are indexed by $j=4...19$.

The distribution of a cohort across the $j$ states at any age $i>0$ is given by the $j$-dimensional column vector:

$$\begin{aligned} s\left( i \right)=\Phi\left( i-1 \right)*s\left( 0 \right).\#\left( 5 \right) \end{aligned}$$

We assume that everyone dies immediately after the 100^th^ birthday, which is equivalent to assuming that the first row of the $\varphi(100)$ matrix is the unit vector.

***1.2. Model parametrization***

*1.2.1. Life tables*

We parametrize background mortality using the National Life Tables for 2013–2015 from the United Kingdom (UK) Office for National Statistics (ONS) (Table S1.1).^1^ For every age $i=0,\ldots,99$, we compute the probability of dying from non-meningococcal serogroup B (MenB)-related causes between $i$ and $i+1$ as the following function of survivorship values:

$$\begin{aligned} p_{d}\left( i \right)=1-\frac{l_{i+1,m}+l_{i+1,f}}{l_{i,m}+l_{i,f}}\#\left( 6 \right) \end{aligned}$$

**Table S1.1. Life Table survivorship functions from the ONS^1^**

| Age (years) | Male (count) | Female (count) | Age (years) | Male (count) | Female (count) | Age (years) | Male (count) | Female (count) |
| --- | --- | --- | --- | --- | --- | --- | --- | --- |
| 0 | 100000 | 100000 | **35** | 98297 | 98963 | **70** | 80540 | 86956 |
| 1 | 99576 | 99654 | **36** | 98196 | 98905 | **71** | 78974 | 85827 |
| 2 | 99543 | 99628 | **37** | 98090 | 98841 | **72** | 77276 | 84597 |
| 3 | 99527 | 99615 | **38** | 97974 | 98775 | **73** | 75363 | 83233 |
| 4 | 99515 | 99603 | **39** | 97841 | 98701 | **74** | 73328 | 81710 |
| 5 | 99505 | 99595 | **40** | 97705 | 98619 | **75** | 71107 | 80050 |
| 6 | 99496 | 99588 | **41** | 97553 | 98529 | **76** | 68758 | 78260 |
| 7 | 99487 | 99581 | **42** | 97390 | 98435 | **77** | 66254 | 76301 |
| 8 | 99478 | 99573 | **43** | 97218 | 98330 | **78** | 63607 | 74186 |
| 9 | 99470 | 99566 | **44** | 97038 | 98216 | **79** | 60764 | 71894 |
| 10 | 99462 | 99560 | **45** | 96838 | 98091 | **80** | 57763 | 69353 |
| 11 | 99452 | 99553 | **46** | 96620 | 97953 | **81** | 54508 | 66543 |
| 12 | 99443 | 99547 | **47** | 96392 | 97809 | **82** | 51106 | 63510 |
| 13 | 99433 | 99541 | **48** | 96145 | 97652 | **83** | 47470 | 60213 |
| 14 | 99421 | 99530 | **49** | 95887 | 97481 | **84** | 43698 | 56678 |
| 15 | 99410 | 99519 | **50** | 95600 | 97296 | **85** | 39836 | 52854 |
| 16 | 99394 | 99505 | **51** | 95292 | 97087 | **86** | 35838 | 48841 |
| 17 | 99373 | 99490 | **52** | 94962 | 96863 | **87** | 31854 | 44668 |
| 18 | 99344 | 99475 | **53** | 94611 | 96618 | **88** | 27923 | 40326 |
| 19 | 99304 | 99455 | **54** | 94230 | 96350 | **89** | 24066 | 35952 |
| 20 | 99257 | 99434 | **55** | 93809 | 96062 | **90** | 20401 | 31541 |
| 21 | 99212 | 99415 | **56** | 93351 | 95739 | **91** | 16967 | 27176 |
| 22 | 99164 | 99394 | **57** | 92853 | 95393 | **92** | 13853 | 23044 |
| 23 | 99118 | 99373 | **58** | 92297 | 95013 | **93** | 11012 | 19119 |
| 24 | 99065 | 99351 | **59** | 91690 | 94610 | **94** | 8504 | 15466 |
| 25 | 99013 | 99329 | **60** | 91021 | 94167 | **95** | 6447 | 12228 |
| 26 | 98956 | 99305 | **61** | 90288 | 93675 | **96** | 4746 | 9462 |
| 27 | 98896 | 99277 | **62** | 89504 | 93141 | **97** | 3427 | 7156 |
| 28 | 98834 | 99250 | **63** | 88645 | 92564 | **98** | 2380 | 5236 |
| 29 | 98772 | 99219 | **64** | 87715 | 91935 | **99** | 1587 | 3705 |
| 30 | 98705 | 99185 | **65** | 86710 | 91268 | **100** | 1031 | 2527 |
| 31 | 98634 | 99147 | **66** | 85642 | 90541 |  |  |  |
| 32 | 98560 | 99106 | **67** | 84526 | 89775 |  |  |  |
| 33 | 98475 | 99063 | **68** | 83309 | 88928 |  |  |  |
| 34 | 98390 | 99016 | **69** | 81995 | 87979 |  |  |  |

ONS Office of National Statistics

*1.2.2. Incidence and case fatality rates*

For the base case, we use average MenB incidence data from 2000 to 2014 (prior to the introduction of the four-component serogroup B meningococcal [4CMenB] vaccination) from Public Health England (Table S1.2a)^2^ and for the sensitivity analysis, we use MenB incidence rates from the European Centre for Disease Prevention and Control (ECDC) surveillance data for 2014 (Table S1.2b).^3^ These incidence rates give us $n_{novax}(i)$, the number of cases per 100000 non-vaccinated of age $i$.

The probability of infection in the non-vaccinated is given by:

$$\begin{aligned} p_{inf}\left( i \right)=1-e^{-\frac{n_{novax}(i)}{100,000}},\#\left( 7 \right) \end{aligned}$$

and the probability in the vaccinated follows the same expression, except that it uses $n_{vax}(i)$, the number of cases per 100000 vaccinated of age $i$.

We use case fatality rates based on data from Ladhani et al. (2016)^4^ and Shigematsu et al. (2002)^5^ (Table S1.2c).

**Table S1.2a. MenB incidence rates, UK, 2000–2014^2^**

| Age | Annual no. of lab reported MenB cases | Mid-year population 2015 | Incidence rate (cases per 100,000) | Age | Annual no. of lab reported MenB cases | Mid-year population 2015 | Incidence rate (cases per 100,000) | Age | Annual no. of lab reported MenB cases | Mid-year population 2015 | Incidence rate (cases per 100,000) |
| --- | --- | --- | --- | --- | --- | --- | --- | --- | --- | --- | --- |
| 0 | 260 | 662977 | 39.22 | **34** | 2 | 748757 | 0.27 | **68** | 2 | 682834 | 0.29 |
| 1 | 83 | 670993 | 12.40 | **35** | 2 | 749992 | 0.27 | **69** | 2 | 525701 | 0.38 |
| 2 | 83 | 688932 | 12.05 | **36** | 2 | 719287 | 0.28 | **70** | 2 | 508499 | 0.39 |
| 3 | 83 | 712587 | 11.65 | **37** | 2 | 670798 | 0.30 | **71** | 2 | 504922 | 0.40 |
| 4 | 83 | 699191 | 11.87 | **38** | 2 | 660061 | 0.30 | **72** | 2 | 467555 | 0.43 |
| 5 | 13 | 687611 | 1.89 | **39** | 2 | 670780 | 0.30 | **73** | 2 | 415572 | 0.48 |
| 6 | 13 | 677724 | 1.92 | **40** | 2 | 683903 | 0.29 | **74** | 2 | 372072 | 0.54 |
| 7 | 13 | 683397 | 1.90 | **41** | 2 | 694559 | 0.29 | **75** | 2 | 385501 | 0.52 |
| 8 | 13 | 661182 | 1.97 | **42** | 2 | 724677 | 0.28 | **76** | 2 | 380304 | 0.53 |
| 9 | 13 | 647549 | 2.00 | **43** | 2 | 754561 | 0.27 | **77** | 2 | 367100 | 0.54 |
| 10 | 13 | 620073 | 2.10 | **44** | 2 | 778754 | 0.26 | **78** | 2 | 345778 | 0.58 |
| 11 | 13 | 609790 | 2.13 | **45** | 2 | 759786 | 0.26 | **79** | 2 | 325545 | 0.61 |
| 12 | 13 | 592130 | 2.20 | **46** | 2 | 777437 | 0.26 | **80** | 2 | 305377 | 0.65 |
| 13 | 13 | 581609 | 2.24 | **47** | 2 | 776611 | 0.26 | **81** | 2 | 278932 | 0.72 |
| 14 | 13 | 596693 | 2.18 | **48** | 2 | 787611 | 0.256 | **82** | 2 | 261228 | 0.77 |
| 15 | 22 | 612676 | 3.59 | **49** | 2 | 787915 | 0.25 | **83** | 2 | 249194 | 0.80 |
| 16 | 22 | 631635 | 3.48 | **50** | 2 | 791066 | 0.25 | **84** | 2 | 231569 | 0.86 |
| 17 | 22 | 641107 | 3.43 | **51** | 2 | 783305 | 0.26 | **85** | 2 | 210446 | 0.95 |
| 18 | 22 | 661031 | 3.33 | **52** | 2 | 765857 | 0.26 | **86** | 2 | 184425 | 1.08 |
| 19 | 22 | 666840 | 3.30 | **53** | 2 | 748704 | 0.27 | **87** | 2 | 160779 | 1.24 |
| 20 | 9 | 673761 | 1.34 | **54** | 2 | 722068 | 0.28 | **88** | 2 | 141595 | 1.41 |
| 21 | 9 | 701685 | 1.28 | **55** | 2 | 692374 | 0.29 | **89** | 2 | 123076 | 1.63 |
| 22 | 9 | 712095 | 1.26 | **56** | 2 | 676158 | 0.30 | **90** | 2 | 104440 | 1.91 |
| 23 | 9 | 740862 | 1.21 | **57** | 2 | 661089 | 0.30 | **91** | 2 | 87880 | 2.28 |
| 24 | 9 | 763854 | 1.18 | **58** | 2 | 635797 | 0.31 | **92** | 2 | 73340 | 2.72 |
| 25 | 2 | 754595 | 0.27 | **59** | 2 | 612904 | 0.33 | **93** | 2 | 60730 | 3.29 |
| 26 | 2 | 749216 | 0.27 | **60** | 2 | 590280 | 0.34 | **94** | 2 | 48860 | 4.09 |
| 27 | 2 | 759356 | 0.26 | **61** | 2 | 591581 | 0.34 | **95** | 2 | 34790 | 5.75 |
| 28 | 2 | 742544 | 0.27 | **62** | 2 | 583991 | 0.34 | **96** | 2 | 21250 | 9.4 |
| 29 | 2 | 752257 | 0.27 | **63** | 2 | 567596 | 0.35 | **97** | 2 | 13600 | 14.71 |
| 30 | 2 | 754383 | 0.27 | **64** | 2 | 571273 | 0.35 | **98** | 2 | 10070 | 19.86 |
| 31 | 2 | 737768 | 0.27 | **65** | 2 | 582595 | 0.34 | **99** | 2 | 20020 | 9.99 |
| 32 | 2 | 744176 | 0.27 | **66** | 2 | 596441 | 0.33 |  |  |  |  |
| 33 | 2 | 742944 | 0.27 | **67** | 2 | 629564 | 0.32 |  |  |  |  |

lab, laboratory; MenB, meningococcal serogroup B; no, number; UK, United Kingdom

**Table S1.2b. UK MenB incidence rates, 2014, ECDC^3^**

| Age | Incidence (cases per 100,000) |
| --- | --- |
| <1 | 17.443 |
| 1–4 | 4.452 |
| 5–14 | 0.572 |
| 15–24 | 0.856 |
| 25–49 | 0.151 |
| 50–64 | 0.196 |
| ≥65 | 0.302 |

ECDC, European Centre for Disease Prevention and Control; MenB, meningococcal serogroup B; UK, United Kingdom

**Table S1.2c. UK case fatality rates4,5**

| Age | Case fatality rate |
| --- | --- |
| <1 | 3.9 |
| 1–2 | 2.7 |
| 3–10 | 3.0 |
| 11–17 | 3.8 |
| ≥18 | 9.7 |

UK, United Kingdom

*1.2.3. Vaccine efficacy*

We assume a 2+1 dose schedule, with vaccine efficacy (VE) denoted by ${ve}_{1}, {ve}_{2}, and {ve}_{3}$ after the first dose at two months $\left( t_{d1} \right)$, the second dose at four months $\left( t_{d2} \right)$, and booster dose at 12 months $\left( t_{d3} \right),$ respectively; and a mean duration of protection of 33 months^6^ $\left( {dop}_{1} \right)$ for the first dose and 38 months^7^ for the second $\left( {dop}_{2} \right)$ and booster doses $\left( {dop}_{d3} \right)$. We represent this as follows:

$$\begin{aligned} VE\left( i=0 \right)={ve}_{1} * \int_{\frac{t_{d1}}{12}}^{\frac{t_{d2}}{12}} e^{-\left\{ \left[ \frac{1}{\frac{{dop}_{1}}{12}} \right]\left( t-\frac{t_{d1}}{12} \right) \right\}}dt+ {ve}_{2}*\int_{\frac{t_{d2}}{12}}^{\frac{t_{d3}}{12}} e^{-\left\{ \left[ \frac{1}{\frac{{dop}_{2}}{12}} \right]\left( t-\frac{t_{d2}}{12} \right) \right\}}dt,\#\left( 8 \right) \end{aligned}$$

$$VE\left( i\geq1 \right)={ve}_{3}*\int_{i.}^{i+1} e^{-\left\{ \left[ \frac{1}{\frac{{dop}_{3}}{12}} \right]\left( t-i \right) \right\}}dt, \left( 9 \right)$$

where $i$ is the time (years) since vaccination (i.e., $i=0$ is the first 12 months since vaccination).

**2. Lifecycle model**

***2.1. The benchmark perfect capital markets model***

A person of age $i$ living in health state $j$ enjoys a health-augmented period utility of

$$\begin{aligned} v_{j}\left( i \right)=q_{j}(i)*u\left( z_{j}(i) \right),\#\left( 10 \right) \end{aligned}$$

where the function $u\left( z_{j}(i) \right)$ has the following constant-relative-risk-aversion (CRRA) form:

$$\begin{aligned} u\left( z \right)=\frac{z^{1-\frac{1}{\sigma}}-z_{0}^{1-\frac{1}{\sigma}}}{1-\frac{1}{\sigma}}.\#\left( 11 \right) \end{aligned}$$

The composite commodity $z_{j}(i)$ is a constant elasticity of substitution (CES) function of goods and services consumption $c_{j}(i)$ and leisure $l_{j}(i)$:

$$\begin{aligned} \begin{aligned} z_{j}\left( i \right)=\left( {a_{c}}^{\frac{1}{\alpha}}{c_{j}\left( i \right)}^{\frac{\alpha-1}{\alpha}}+{a_{l}}^{\frac{1}{\alpha}}{l_{j}\left( i \right)}^{\frac{\alpha-1}{\alpha}} \right)^{\frac{\alpha}{\alpha-1}} , \end{aligned}\#\left( 12 \right) \end{aligned}$$

where $a_{l}=1-a_{c}$. The health utilities $q_{j}(i)$ are age- and state-specific.

The functional form in $(11)$ is variously called an *isoelastic*, CRRA, or power function. It has two parameters: $\sigma,$ which is the *elasticity of intertemporal substitution* and $z_{0,}$ which is the level of *subsistence* composite consumption. The advantage of this isoelastic function is that it is a simple way to incorporate a person’s elasticity of intertemporal substitution. This is a person’s tolerance for volatility of consumption over time. The higher the value of $\sigma$, the higher the tolerance for variability, and as $\sigma\to\infty$ the person becomes indifferent to such volatility. As $\sigma\to0$, the preference for stable consumption gets stronger. We shall see that willingness to pay (WTP) for mortality and morbidity risk reduction is sensitive to the magnitude of this tolerance for volatility.

The level of subsistence consumption $z_{0}$ is the level of composite consumption at which a person would be indifferent between living and dying. All economic models that value mortality risk reductions are confronted with the need to specify the utility of death. The approach we take, and that is taken by others,^8^ is that we assume the utility of death to be constant, and that there is some level of consumption $z_{0}$ needed to make life worth living, so that if consumption were to fall below that level the person would rather be dead. Note that the functional form in $(11)$ implies that $u\left( z_{0} \right)=0$. That is, equation $(11)$ implicitly normalizes moment utility so that the utility of death equals zero.

The functional form in $(12)$ implies that the composite consumption good $z$ is a CES aggregate of consumption and non-market time where $a_{l}$ and $a_{c}$ are constants and $\alpha$ is the elasticity of substitution between goods and services and non-market time. The elasticity of substitution $z$ is a measure of how substitutable (or interchangeable) the person finds $c$ and $l$. The higher this elasticity, the more substitutable these are and the more sensitive will relative quantities (i.e., $\frac{c}{l}$) be to changes in their relative price.

A CES aggregate in $(12)$ is a special case of homothetic preferences, which in turn reflects a preference for consuming goods and services and non-market time in fixed proportions, where the fixed proportion depends on the relative price of goods and services on the one hand and non-market time on the other. In other words, for a given relative price of goods and services and non-market time, CES preferences imply that the individual’s optimal ratio of quantities consumed of goods and services and non-market time will be fixed regardless of the scale of those quantities, though changing the relative price would change that ratio. It is a compact way to allow an individual’s choices over goods and services and non-market time to be sensitive to their relative price (an important phenomenon), while avoiding the complexity of scale effects (a lesser concern).

Equations $(11)$ and $(12)$ allow us to express moment utility as a function of only one composite consumption good, so long as this person tends to prefer the two forms of consumption $c$ and $l$ in fixed proportions (which proportions can nevertheless vary as a function of relative price). Equation $(11)$ tells us that a person’s preferences for the composite consumption good $z$ are such that (i) more of $z$ is always better (because $u_{z}=\frac{\partial u}{\partial z}>0$), (ii) the person prefers stable lifetime trajectories of $z$ over volatile ones, and (iii) life is only worth living when composite consumption exceeds some subsistence threshold. Equation (10) says that health utility and composite consumption are natural complements, i.e., the partial derivative of $v_{j}$ with respect to $q_{j}$ is a positive function of $z_{j}$ and vice versa. Stated differently, health and consumption are mutually enhancing in that the healthier an individual, the higher the benefit from extra consumption and vice versa.

A useful expression in what follows is the age- and health-state-specific marginal utility of consumption:

$\begin{aligned} v_{cj}\left( i \right)\equiv\frac{\partial v\left( v_{j}\left( i \right) \right)}{\partial c_{j}\left( i \right)}=q_{j}\left( i \right)*\left( z_{j}\left( i \right) \right)^{\frac{1}{\alpha}-\frac{1}{\sigma}}*a_{c}^{\frac{1}{\alpha}}*\left( c_{j}\left( i \right) \right)^{-\frac{1}{\alpha}}.\#\left( 13 \right) \end{aligned}$We collected the $j$ health-state-specific $v_{j}\left( i \right)$ into a single $j$-dimensional row vector:

$$\begin{aligned} v\left( i \right)=\left\{ v_{1}\left( i \right)\ldots v_{19}\left( i \right) \right\}.\#\left( 14 \right) \end{aligned}$$

The representative member of the cohort maximizes expected discounted lifetime utility (a scalar):

$$\begin{aligned} U\left( a \right)=\sum_{i=a}^{100} \frac{v\left( i \right)s\left( i \right)}{{(1+\rho)}^{i-a}}\#\left( 15 \right) \end{aligned}$$

subject to the lifetime budget constraint requiring that the expected present discounted value of lifetime consumption not exceed the expected present discounted value of lifetime earnings and initial wealth:

$$\begin{aligned} A\left( a \right)+\sum_{i=a}^{100} \frac{s\left( i \right)*\left[ y\left( i \right)-c\left( i \right) \right]}{{(1+r)}^{i-a}}=0.\#\left( 16 \right) \end{aligned}$$

In this budget constraint, the scalar $A\left( a \right)$ is initial wealth (upon model entry), $y\left( i \right)$ and $c\left( i \right)$ are $j$-dimensional row vectors containing annual earnings and consumption in each of the $j$ health states (though earnings and consumption are zero for the death state $j=0$). The representative element in the annual earnings vector has the following additional structure:

$$\begin{aligned} y_{j}\left( i \right)=w_{j}\left( i \right)\left[ T-l_{j}\left( i \right) \right]\#\left( 17 \right) \end{aligned}$$

This says that annual earnings at age $i$ and state $j$ depends on the hourly wage $w_{j}\left( i \right)$ and annual hours working for pay $\left[ T-l_{j}\left( i \right) \right],$ which in turn depends on annual time endowment $T$ and non-market time $l_{j}\left( i \right)$ (which is the sum of unpaid work and leisure time). Note that the hourly wage $w_{j}\left( i \right)$ depends on both age $i$ and health state $j$. The hourly wages of those with MenB-related disabilities are a fraction of those of uninfected adults.

The above budget constraint in equation (16) assumes “perfect capital markets.” There are three aspects to this assumption worth explicitly spelling out. First is the assumption of perfect credit markets that allow a person to shift resources freely over time, for example saving any current income in excess of current consumption or borrowing to finance current consumption in excess of current income, all at the rate $r$. Second is a perfect annuity market that effectively allows a person to insure consumption against longevity risk through the present purchase of an annuity that makes future payouts (from which future consumption can be financed)^8^ so long as the person remains alive. We can imagine that there is a company to which this individual relinquishes all lifetime wealth and income, and in return the company will pay out at each moment $i$ the relevant annuity $c$ and shall make such payouts so long as the person lives. The third aspect is perfect disability insurance: the individual effectively has access to an insurance policy that pays out when disabled and for which the individual pays a premium when non-disabled. If we might imagine a single company that provides credit, annuities, and disability insurance, the budget constraint is simply the break-even condition for this company.

In the absence of vaccination (i.e., when the cumulative matrix $\Phi\left( i \right)$ is computed using $n_{novax}(i)$), the representative member of the cohort maximized utility subject to the budget constraint, which can be represented by maximizing the following Lagrangian:

$$\begin{aligned} L=\max_{c_{j}\left( i \right),l_{j}\left( i \right),\mu} \sum_{i=a}^{100} \frac{v\left( i \right)s\left( i \right)}{{(1+\rho)}^{i-a}}+\mu\left[ A\left( a \right)-\sum_{i=a}^{100} \frac{s\left( i \right)*\left[ y\left( i \right)-c\left( i \right) \right]}{{(1+r)}^{i-a}} \right].\#\left( 18 \right) \end{aligned}$$

Note that in this problem, the individual’s choice variables are age- and state-specific consumption and non-market time, represented by $c_{j}\left( i \right)$ and $l_{j}\left( i \right)$ (see equation (17)). The following are exogenous (i.e., taken by the individual as fixed: the cohort distribution $s(i)$, initial wealth $A\left( a \right)$, health utilities $q_{j}(i)$, wages $w_{j}(i)$, the market interest rate $r$, and the preference parameters which include $\sigma,z_{0},\rho,\alpha,a_{c},a_{l}$).

The analytical solution to the maximization problem is given by (for $j\neq1)$:

$$\begin{aligned} c_{j}(i)=\frac{a_{c}}{a_{c}+a_{l}{w_{j}\left( i \right)}^{1-\alpha}}*c_{j}^{F}(i),\#\left( 19 \right) \end{aligned}$$

$$\begin{aligned} l_{j}(i)=\frac{a_{l}{w_{j}\left( i \right)}^{1-\alpha}}{a_{c}+a_{l}{w_{j}\left( i \right)}^{1-\alpha}}*\left( \frac{1}{w_{j}\left( i \right)} \right)*c_{j}^{F}(i),\#\left( 20 \right) \end{aligned}$$

$$\begin{aligned} \mu=\left( \frac{A\left( a \right)+T\sum_{i=a}^{100} \frac{\sum_{j=2}^{19} w_{j}\left( i \right)s_{j}\left( i \right)}{{(1+r)}^{i-a}}}{\sum_{i=a}^{100} {[{(1+r)}^{\sigma-1}/{(1+\rho)}^{\sigma}]}^{\left( i-a \right)}\sum_{j=2}^{19} s_{j}\left( i \right){q_{j}(i)}^{\sigma}{K_{j}(i)}^{\sigma-1}} \right)^{-\frac{1}{\sigma}},\#\left( 21 \right) \end{aligned}$$

$$\begin{aligned} c_{j}^{F}(i)={K_{j}(i)}^{\sigma-1}\left( \left( \frac{1}{q_{j}(i)} \right)*\mu*\left( \frac{1+\rho}{1+r} \right)^{i-a} \right)^{-\sigma},\#\left( 22 \right) \end{aligned}$$

$$\begin{aligned} K_{j}(i)=\left( a_{c}+a_{l}{w_{j}\left( i \right)}^{1-\alpha} \right)^{\frac{1}{\alpha-1}},\#\left( 23 \right) \end{aligned}$$

$$\begin{aligned} z_{j}=c_{j}^{F}*K_{j}\left( i \right).\#\left( 24 \right) \end{aligned}$$

In the above expressions (equations (19) to (24)), $c_{j}^{F}(i)$ is called full consumption and is defined as the sum of consumption $c_{j}(i)$ and the value of non-market time $w_{j}\left( i \right)*l_{j}(i)$. Note the difference between two expressions for composite commodity consumption $(12)$ and $(24)$. Equation $(12)$ defines composite commodity consumption and so is always true. Equation $(24)$ was an optimality condition and so is only true when consumption and non-market time are set optimally according to $(19)$ and $(20)$. Note too that in the equation (21) expression for $\mu$, the elements $w\left( i \right),s\left( i \right),q\left( i \right),K(i)$ are all j-vectors, while $\mu$ is a scalar.

Recall that the above problem and solution presume no vaccination. Given such a solution, vaccination alters the trajectory of the cohort through the Markov model. We can summarize the impact of vaccination in terms of a shift of the distribution of the cohort across the Markov states, from that which occurs in a no-vaccination scenario (which we referred to by the $j$-dimensional column vector $s\left( i \right)$ but which we will now denote $s^{novax}\left( i \right)$ for clarity) to that which occurs in a vaccination scenario, which we denoted $s^{vax}\left( i \right)$. Using the envelope theorem, for small perturbations around the optimum, the impact of vaccination can be estimated by the impact on the Lagrangian of the change $ds$ (see equation $(25)$) in the distribution of the cohort across the Markov states. The impact of this change on the Lagrangian is:

$$\begin{aligned} \Delta L= \sum_{i=a}^{100} \frac{v\left( i \right)ds\left( i \right)}{{(1+\rho)}^{i-a}}+\mu\left[ \sum_{i=a}^{100} \frac{\left[ y\left( i \right)-c\left( i \right) \right]}{{(1+r)}^{i-a}}ds\left( i \right) \right].\#\left( 25 \right) \end{aligned}$$

After some simplification, we get:

$$\begin{aligned} \frac{\Delta L}{\mu}=\left[ \sum_{i=a}^{100} \sum_{j=1}^{19} \left[ \frac{v_{j}(i)}{v_{cj}\left( i \right)}+y_{j}\left( i \right)-c_{j}\left( i \right) \right]ds_{j}\left( i \right)\frac{1}{{(1+r)}^{i-a}} \right]\#\left( 26 \right) \end{aligned}$$

Equation $(26)$ is our fundamental valuation equation. It is a scalar and is denominated in money terms and approximates a representative cohort member’s WTP for vaccination. To understand this equation, it is helpful to take a representative term for a given age $i$ and health state $j$ and to ignore discounting:

$$\begin{aligned} \left[ \frac{v_{j}(i)}{v_{cj}\left( i \right)}+y_{j}\left( i \right)-c_{j}\left( i \right) \right]ds_{j}\left( i \right).\#\left( 27 \right) \end{aligned}$$

Simplifying somewhat, the disease model determines the probability $s_{j}\left( i \right)$ of being in state $j$ at age $i$ while the lifecycle model determines the monetary value (given in the square bracketed term of (27)) of being in state $j$ at age $i$. Vaccination produces value by changing the probabilities of being in the various states, changes denoted by $ds_{j}\left( i \right)$. We can therefore think of $(27)$ as a price times quantity expression, with the price term in square brackets and the quantity term represented by the vaccination-induced change in probability of occupying a particular age-health-state combination.

The monetary value of each state can be broken down to its intrinsic value $\frac{v_{j}\left( i \right)}{v_{cj}\left( i \right)}$ and its instrumental value $y_{j}\left( i \right)-c_{j}\left( i \right)$. The intrinsic value of a state is simply its contribution $v_{j}\left( i \right)$ to the lifetime utility function in $(15)$ but divided by the marginal (health-augmented) utility of consumption $v_{cj}\left( i \right)$ to convert it into monetary terms. The instrumental value of a state is the extent to which being in that state produces resources that can be used for consumption in other states, an extent measured by net savings $y_{j}\left( i \right)-c_{j}\left( i \right)$: being in that state “loosens” the lifetime budget constraint since it allows the realization of the earnings $y_{j}\left( i \right)$ associated with that state, but it “tightens” the lifetime budget constraint because it requires the consumption $c_{j}\left( i \right)$ associated with that state, so the net loosening of the budget constraint associated with being in that state is the difference between the two. We call this monetary value the value of a statistical life year (VSLY) and denote it by:

$$\begin{aligned} {VSLY}_{j}\left( i \right)=\frac{v_{j}\left( i \right)}{v_{cj}\left( i \right)}+y_{j}\left( i \right)-c_{j}\left( i \right).\#\left( 28a \right) \end{aligned}$$

For obvious reasons, ${VSLY}_{1}\left( i \right)=0$, where $j=1$ represents the dead state. One of the first-order conditions of the Lagrangian is given by:

$$\begin{aligned} v_{cj}\left( i \right)=\mu*\left( \frac{1+\rho}{1+r} \right)^{i-a}.\#\left( 28b \right) \end{aligned}$$

Since we assume $\rho=r$, this allows us to rewrite $(28a)$ as:

$$\begin{aligned} {VSLY}_{j}\left( i \right)=\frac{v_{j}\left( i \right)}{\mu}+y_{j}\left( i \right)-c_{j}\left( i \right)\#\left( 28c \right) \end{aligned}$$

***2.2. Children***

Our lifecycle model does not apply well to children, who make no labor-leisure trade-offs. We therefore perform an ad hoc modification of the VSLY formula for children. We define children as aged 0–15 (inclusive) and assume that for all $0\leq i\leq15$ and for all $j>1$ (and using a superscript $c$ to represent children):

$$\begin{aligned} l_{j}^{c}\left( i \right)=l^{c}(i)=T,\#\left( 29 \right) \end{aligned}$$

$$\begin{aligned} y_{j}^{c}\left( i \right)=y^{c}(i)=0.\#\left( 30 \right) \end{aligned}$$

In other words, children do not perform paid work and devote their entire time endowments to non-market activities. We also assume that:

$$\begin{aligned} c_{j}^{c}\left( i \right)=c^{c}(i)=\hat{c}\left( i \right).\#\left( 31 \right) \end{aligned}$$

In other words, consumption is unaffected by disability (parents find some way to finance this, perhaps by reducing their own consumption) and equal in value to levels of consumption observed among children in the general population. Composite commodity consumption is determined by substituting the above expressions into $(12)$ to get:

$$\begin{aligned} z_{j}^{c}\left( i \right)=z^{c}(i)=\left( {a_{c}}^{\frac{1}{\alpha}}{\hat{c}\left( i \right)}^{\frac{\alpha-1}{\alpha}}+{a_{l}}^{\frac{1}{\alpha}}T^{\frac{\alpha-1}{\alpha}} \right)^{\frac{\alpha}{\alpha-1}}.\#\left( 32 \right) \end{aligned}$$

Note that non-market time, earnings, consumption, and composite commodity consumption thus defined are unaffected by disability (i.e., they are invariant across $j=2,\ldots,19$). Health-augmented period utility is:

$$\begin{aligned} v_{j}^{c}\left( i \right)=\frac{q_{j}\left( i \right)}{1-\frac{1}{\sigma}}\left[ {z^{c}(i)}^{1-\frac{1}{\sigma}}-z_{0}^{1-\frac{1}{\sigma}} \right].\#\left( 33 \right) \end{aligned}$$

This varies with respect to $j$ because of the health utility term. Building on $(28c)$, we defined the matrix (representing 19 health states and 16 ages from 0–15) for ${VSLY}^{c}$ for children with representative element:

$$\begin{aligned} {VSLY}_{j}^{c}\left( i \right)=\frac{v_{j}^{c}\left( i \right)}{\mu}-c^{c}\left( i \right).\#\left( 34 \right) \end{aligned}$$

Note that ${VSLY}_{j}^{c}\left( i \right)$ varies with respect to $j$ solely because of the health utility term. Note, too, that the first row of the ${VSLY}^{c}$ matrix, which corresponds to the dead state, consists of zeros.

***2.3. Perfect capital markets: a data-driven approach***

If we were fully confident in our model’s predictions of how full consumption varied over the lifecycle, we would use equation $(22)$ and the underlying parameters of the model to compute full consumption across all states $j$ and ages $i$. We would compute full consumption over the lifecycle in the uninfected state using:

$$\begin{aligned} c_{u}^{F}(i)={K_{u}(i)}^{\sigma-1}\left( \left( \frac{1}{q_{j}(i)} \right)*\mu*\left( \frac{1+\rho}{1+r} \right)^{i-a} \right)^{-\sigma}.\#\left( 35 \right) \end{aligned}$$

Note that this equation is a specific version of $\left( 22 \right),$ where we have replaced the subscript $j$ with the sub-script $u$ representing the uninfected state. However, we find that equation $(22)$ does not convincingly track data on lifecycle full consumption $\hat{c}_{u}^{F}(i)$ (see equation $(47)$ for the construction of such data). We believe this is in part because our budget constraints facilitate borrowing in early adult life and annuitization of consumption in late life, leading to model-predicted consumption that is higher in early adult life and in late life than is realistically possible.

Thus, instead of using the model-generated $c_{u}^{F}(i)$ defined by $\left( 35 \right)$, we use the data-based estimate $\hat{c}_{u}^{F}(i)$ defined by $(47)$ (see details in section 3.8.1). We then proceed as follows: for any $j\neq u$, we use $(22)$ to get the following expression for $c_{j}^{F}\left( i \right)$ conditional on $c_{u}^{F}(i)$ and replace $c_{u}^{F}(i)$ with $\hat{c}_{u}^{F}(i)$:

$$\begin{aligned} \frac{c_{j}^{F}\left( i \right)}{\hat{c}_{u}^{F}(i)}=\left( \frac{K_{j}\left( i \right)}{K_{u}\left( i \right)} \right)^{\sigma-1}\left( \frac{q_{u}\left( i \right)}{q_{j}\left( i \right)} \right)^{-\sigma}\to c_{j}^{F}\left( i \right)=\hat{c}_{u}^{F}(i)\left( \frac{K_{j}\left( i \right)}{K_{u}\left( i \right)} \right)^{\sigma-1}\left( \frac{q_{u}\left( i \right)}{q_{j}\left( i \right)} \right)^{-\sigma}\#\left( 36 \right) \end{aligned}$$

For any $j\neq u,$ $\left( 36 \right)$ provides us with a *data-based* estimate of $c_{j}^{F}\left( i \right)$. We can use these data-based estimates of $c_{j}^{F}\left( i \right)$ in equations $(19)$ and $(20)$ to give us data-based estimates of consumption and non-market time (reflecting the assumption that within-period consumption-leisure choices are made according to the model). We can in turn use these in equations $(17)$ and $\left( 24 \right)$ to give us data-based estimates of earnings and composite commodity consumption, and in equation $(10)$ to get age- and health-state-specific utility $v_{j}(i)$. All the above allow us to populate the expression for ${VSLY}_{j}(i)$ in $(28c)$. We refer to the ${VSLY}_{j}(i)$ thus calculated as “data-based perfect capital markets” values. We refer to the 19-by-85 (19 health states and 85 ages from 16 to 100) matrix thus calculated as the ${VSLY}^{pcm}$ matrix. As before, the first row of this matrix corresponding to the dead state consists of zeros.

***2.4. Imperfect capital markets***

The perfect capital markets model, even in its data-driven version, assumes that an individual can set levels of consumption, non-market time, and full consumption in disabled states optimally relative to their levels in the uninfected state. In particular, it doesn’t allow any adverse effects of disability on productivity and earnings to hinder individuals from complying with the optimality conditions $(19)$, $(20)$, and $(22)$: disability insurance implicitly allows individuals to satisfy these even in face of the adverse effects of disability on productivity and earnings.

We now provide ad hoc estimates of ${VSLY}_{j}(i)$ in the absence of disability insurance. Meyer and Mok (2019)^9^ provide United States (US)-based evidence that the percentage decline in consumption resulting from disability is roughly one-third of the earnings decline from disability. Consumption does not fall one-for-one with earnings because, for example, other household members may raise their earnings, or the household may receive gifts or disability insurance payments or benefits. Using this evidence and UK-based evidence from Longhi (2017)^10^ and the UK Green Paper on Work, Health, and Disability (2016),^11^ we compute a variable called “consumption gap” (Table 1) representing the percentage decline in consumption resulting from disability:

$$\begin{aligned} \frac{c_{j}\left( i \right)}{c_{u}\left( i \right)}\approx{Consumption gap}_{j}\to c_{j}\left( i \right)\approx{Consumption gap}_{j}*c_{u}\left( i \right)\#\left( 37 \right) \end{aligned}$$

As in the perfect capital markets case, we compute $c_{u}\left( a \right)$ and $l_{u}\left( a \right)$ on the basis $(19)$ and $(20)$ using $\hat{c}_{u}^{F}(i)$ instead of $c_{u}^{F}(i)$ in these equations. In other words, we follow the data-driven approach to fixing full consumption when uninfected but assume that conditional on such full consumption, the individual is still able to optimize its division into consumption and non-market-time. After all, in the uninfected state, it is less problematic to assume that the individual can optimize thusly. Given $c_{u}\left( a \right)$ and the “consumption gap,” we use $(19)$ to generate $c_{j}\left( i \right)$ for $j>2$. We assume that the impact of disability on hours worked is measured by our data on the “hours worked gap” in Table 1:

$$\begin{aligned} \frac{T-l_{j}\left( i \right)}{T-l_{u}\left( i \right)}\approx{Hours worked gap}_{j}\to l_{j}\left( i \right)=T-{Hours worked gap}_{j}*\left( T-l_{u}\left( i \right) \right)\#\left( 38 \right) \end{aligned}$$

Thus, given $l_{u}\left( a \right)$ and the “hours worked gap” data in Table 1, we use $(20)$ to compute $l_{j}\left( i \right)$ for $j>2$. The above in turn allow us to compute earnings and composite commodity consumption using $(17)$ and $(12)$, the latter of which in turn allows the computation of health-augmented utility using $(10)$ and $(11)$.

A question arises whether to define $VSLY$ in the imperfect capital markets case using $(28a)$ or $(28c)$. When the individual has access to disability insurance (as implied by the budget constraint $(16)$) and is following the optimality conditions of the model (in particular equations $\left( 19 \right)-(24)$ and $(28b)$), then $(28a)$ or $(28c)$ are equivalent and so yield identical results. However, the purpose of the imperfect capital markets scenario is that such disability insurance may not be available and that the individual may therefore be unable to comply with $\left( 19 \right)-(24)$ and $(28b)$. Under imperfect capital markets scenario, therefore, marginal (health-augmented) utility of consumption will be defined by $(13)$ (because it is derived from $(12)$, which recall is always true). Thus, in our baseline we build on equation $(28c)$. We therefore define the 19-by-85 matrix ${VSLY}^{icm}$ with representative element:

$$\begin{aligned} {{VSLY}_{j}}^{icm}\left( i \right)=\frac{v_{j}\left( i \right)}{v_{cj}\left( i \right)}+y_{j}\left( i \right)-c_{j}\left( i \right)\#\left( 39 \right) \end{aligned}$$

In sensitivity analysis, we build on $(28a)$ instead of $(28c)$, and use $\mu$ in the denominator of $(39)$ instead of $v_{cj}\left( i \right)$.

**3. Lifecycle model – detailed assumptions and inputs**

***3.1. Parental spillovers***

Based on Al-Janabi et al. (2016),^12^ we assume that having a permanently disabled (PD) child raises the risk of parental depression by 17 percentage points.

We define:

$$\begin{aligned} s_{PD}^{novax}\left( i \right)=\sum_{j=4}^{19} s_{j}^{novax}\left( i \right),\#\left( 40 \right) \end{aligned}$$

to equal the sum across all PD states of the shares of an unvaccinated cohort in those states. Define $s_{PD}^{vax}\left( i \right)$ as the parallel quantity in a vaccinated cohort. Thus, the impact of vaccination on the probability of being in a permanently disabled state is:

$$\begin{aligned} {\delta s_{PD}\left( i \right)=s}_{PD}^{no-vax}\left( i \right)-s_{PD}^{vax}\left( i \right)>0.\#\left( 41 \right) \end{aligned}$$

Let $i_{p}=i+\Delta$ be the parent’s (“$p$”) age, where $\Delta$ is the age differential between parent and child. For a parent of age $i_{p}$ (we assume a parental age at birth of $\Delta=32$)^13^ whose child has a permanent disability, the gain in avoiding anxiety and depression is:

$$\begin{aligned} \delta VSLY\left( i_{p} \right)\equiv{{VSLY}_{u}}^{icm}\left( i_{p} \right)-{{VSLY}_{j=d}}^{icm}\left( i_{p} \right).\#\left( 42 \right) \end{aligned}$$

In this expression, ${{VSLY}_{u}}^{icm}(i_{p})$ and ${{VSLY}_{j=d}}^{icm}(i_{p})$ are the $VSLY$ in the uninfected and depressed states, respectively, at age $i_{p}$ from the ${VSLY}^{icm}$ matrix. The parental spillover benefits (PSB) at each age can be summarized by a 101-element row vector $PSB$ with representative element:

$$\begin{aligned} PSB\left( i \right)=\delta s_{PD}\left( i \right)*0.17*\delta VSLY\left( i_{p} \right)*\frac{1-s_{1}^{novax}\left( i_{p} \right)}{1-s_{1}^{novax}\left( \Delta\right)}.\#\left( 43 \right) \end{aligned}$$

In this expression, $s_{1}^{novax}\left( i_{p} \right)$ represents the percentage of an unvaccinated cohort (we assumed the parents enjoying the spillovers are themselves unvaccinated) that is in the dead state at age $i_{p}$. Note that when $i=0$, then the $i_{p}=\Delta$ and the ratio term equals 1. When $i_{p}>100$, then we impose $s_{1}^{novax}\left( i_{p} \right)=1$, reflecting our assumption that no one lives past the 100^th^ birthday, so the ratio term equals 0. As parents age between $\Delta$ and $100$, the ratio term declines from 1 to 0, reflecting the fact that parents die as they age so that the number of beneficiaries from household spillovers declines as parents age. ^13-15^

***3.2. Acute phase disability***

The lifecycle model thus far fails to account for the acute phase of disability (APD), which can be severe. During the acute phase of all non-fatal infections, we assume that health-related quality of life (HRQoL) plummets to 0.065 at the moment of infection and recovers linearly to its steady state level by the fiftieth day.^16^ This implies that the health utility loss over the year immediately following infection at age $i$ resulting in disability state $j$ can be approximated by a $j-$dimensional vector $APD\left( i \right)$ with representative element:

$$\begin{aligned} {dq}_{j}^{APD}(i)=\frac{1}{2}*\left( q_{j}\left( i \right)-0.065 \right)*\left( \frac{50}{365.25} \right)\#\left( 44 \right) \end{aligned}$$

We also assume that one parent loses 7.14 days of work during the acute phase^15^ resulting in lost earnings represented by a 101-vector $PLE$ (for “parental lost earnings”) with representative scalar element:

$$\begin{aligned} PLE\left( i \right)=7.14*\frac{\hat{y}_{u}\left( i+\Delta\right)}{365.25}.\#\left( 45 \right) \end{aligned}$$

Building on $(45)$, we approximate the WTP for avoiding acute phase disability with the 19-by-101-dimensional matrix ${WTP}^{ADP}$ with representative element:

$$\begin{aligned} {WTP}_{j}^{APD}\left( i \right)=\frac{{dq}_{j}^{APD}\left( i \right)*u\left( \hat{c}_{u}^{F}\left( i \right)*K_{j}\left( i \right) \right)}{\mu}+PLE\left( i \right).\#\left( 46 \right) \end{aligned}$$

***3.3. Health utilities in the uninfected state***

We obtain general population HRQoL from Kind et al. (1999)^16^ (Table S1.3).

**Table S1.3. Age-specific HRQoL in the UK general population^16^**

| Age | HRQoL |
| --- | --- |
| <25 | 0.94 |
| 25–34 | 0.93 |
| 35–44 | 0.91 |
| 45–54 | 0.85 |
| 55–64 | 0.80 |
| 65–74 | 0.78 |
| ≥75 | 0.73 |

HRQoL, health-related quality of life; UK, United Kingdom

***3.4 Calibrating the lifecycle model***

*3.4.1. Currencies,* *consumer price indices and exchange rates.*

We translate all currency units into 2018 British Pound Sterling (GBP) (this choice of year avoid non-representative COVID-induced price dynamics). For inflation adjustments, we use an UK ONS consumer price index (CPI)^17^ (Table S1.4).

**Table S1.4. Consumer price indices ^17^**

|  | UK CPI |
| --- | --- |
| 2005 | 78.1 |
| 2006 | 79.9 |
| 2007 | 81.8 |
| 2008 | 84.7 |
| 2009 | 86.6 |
| 2010 | 89.4 |
| 2011 | 93.4 |
| 2012 | 96.1 |
| 2013 | 98.5 |
| 2014 | 100.0 |
| 2015 | 100.0 |
| 2016 | 100.7 |
| 2017 | 103.4 |
| 2018 | 105.9 |

CPI, consumer price index; UK, United Kingdom

We obtain data on age-specific annual consumption, hourly wages, annual earnings, and daily non-market time, all in the uninfected state, which we denoted by $\hat{c}_{u}\left( i \right),\hat{w}_{u}\left( i \right),\hat{y}_{u}\left( i \right),\hat{l}_{u}\left( i \right),$ respectively. We define:

$$\begin{aligned} \hat{c}_{u}^{F}\left( i \right)=\hat{c}_{u}\left( i \right)+\hat{w}_{u}\left( i \right)*\hat{l}_{u}\left( i \right),\#\left( 47 \right) \end{aligned}$$

$$\begin{aligned} \hat{y}_{u}^{F}\left( i \right)=\hat{y}_{u}\left( i \right)+\hat{w}_{u}\left( i \right)*\hat{l}_{u}\left( i \right).\#\left( 48 \right) \end{aligned}$$

Further details on the calibration of the parameters are given below.

*3.4.2. Hourly wages*

We obtain hourly wages $\hat{w}_{u}(i)$ in 2018 GBP from the Annual Survey of Hours and Earnings (Table S1.5).^18^ Since hourly wages are unavailable for ages below 16, and since such wages are needed to value non-market time, we project hourly wages at 16 down to age 0.

**Table S1.5. Hourly wages, UK, 2018, in 2018 GBP^18^**

| Age | Hourly wages |
| --- | --- |
| 16–17 | 4.98 |
| 18–21 | 8.45 |
| 22–29 | 12.05 |
| 30–39 | 15.45 |
| 40–49 | 16.53 |
| 50–59 | 15.09 |
| ≥60 | 13.26 |

GBP, British Pound Sterling; UK, United Kingdom

*3.4.3. Time use*

We obtain time use from the Office of National Statistics.^19^ We take market time to consist of “working and work-related activities” and non-market time $\hat{l}_{u}(i)$ to consist of unpaid work and leisure.^19^ Unpaid work consists of “household activities, purchasing goods and services, caring for and helping household members, caring for and helping non-household members, organizational, civic, and religious activities, telephone calls, mail, and e-mail;” and leisure consists of “leisure and sports.” We ignore time spent on personal care, eating and drinking, educational activities, and other activities (Table S1.6).

**Table S1.6. Time use, UK, 2018, minutes per day^19^**

|  | Unpaid work | Leisure | Paid work | Other |
| --- | --- | --- | --- | --- |
| 16 to 24 (Men) | 85.2 | 387.4 | 256.6 | 710.8 |
| 25 to 34 (Men) | 130.4 | 301.8 | 310.4 | 697.4 |
| 35 to 44 (Men) | 165.8 | 306.9 | 282.4 | 684.9 |
| 45 to 54 (Men) | 153.4 | 345.0 | 237.9 | 703.7 |
| 55 to 64 (Men) | 145.9 | 378.9 | 203.4 | 711.8 |
| 65 and over (Men) | 142.4 | 455.2 | 34.0 | 808.3 |
|  |  |  |  |  |
| 16 to 24 (Women) | 124.1 | 328.1 | 226.8 | 761.0 |
| 25 to 34 (Women) | 249.6 | 271.2 | 198.7 | 720.5 |
| 35 to 44 (Women) | 273.1 | 279.8 | 174.0 | 713.1 |
| 45 to 54 (Women) | 209.4 | 294.4 | 192.8 | 743.4 |
| 55 to 64 (Women) | 214.9 | 337.2 | 133.8 | 754.1 |
| 65 and over (Women) | 204.9 | 409.8 | 10.6 | 814.8 |

UK, United Kingdom

*3.4.4. Consumption and labor income*

We obtain consumption $\hat{c}_{u}\left( i \right)$ as the sum of private and public consumption, other than health or education, from the National Transfer Accounts Project.^19^ This database disaggregates per capita consumption into private and public consumption, where public consumption includes, among other things, consumption of public goods like roads and public infrastructure. It also disaggregates consumption by whether consumption is devoted to education, health, and everything else. However, we exclude consumption expenditures on education on health on grounds that these are forms of human capital investment whose returns are already reflected in earnings (in the case of education expenditures) and survival and HRQoL (in the case of health expenditures). Thus, failing to exclude these education and health expenditures would be a form of double counting. We also obtain labor income $\hat{y}_{u}(i)$from the National Transfer Accounts Project from 2012 GBP^19^ (Table S1.7).

**Table S1.7. Labor income (LY), public (PUB) and private (PRI) consumption other than health and education for the UK (2012 GDP)^19^**

|  | GBP | | |  | GBP | | |  | GBP | | |
| --- | --- | --- | --- | --- | --- | --- | --- | --- | --- | --- | --- |
| Age | **LY** | **PUB** | **PRI** | **Age** | **LY** | **PUB** | **PRI** | **Age** | **LY** | **PUB** | PRI |
| 0 | 2.87E-12 | 2061.28 | 5119.33 | **31** | 25372.19 | 2384.66 | 14350.86 | **62** | 12340.99 | 2641.52 | 16458.11 |
| 1 | 9.55E-11 | 2061.28 | 5140.98 | **32** | 25862.75 | 2399.00 | 14160.90 | **63** | 10584.55 | 2642.71 | 16414.25 |
| 2 | 2.89E-09 | 2061.28 | 5179.12 | **33** | 26236.46 | 2409.59 | 14073.97 | **64** | 8945.04 | 2639.29 | 16381.53 |
| 3 | 7.65E-08 | 2061.28 | 5251.62 | **34** | 26453.43 | 2417.69 | 14134.71 | **65** | 7360.65 | 2634.62 | 16310.70 |
| 4 | 1.71E-06 | 2061.28 | 5380.31 | **35** | 26584.55 | 2424.61 | 14302.65 | **66** | 5824.42 | 2633.46 | 16186.61 |
| 5 | 3.13E-05 | 2061.28 | 5577.82 | **36** | 26787.02 | 2431.17 | 14484.55 | **67** | 4384.31 | 2642.30 | 16030.83 |
| 6 | 0.00 | 2061.28 | 5837.06 | **37** | 27154.59 | 2438.61 | 14601.42 | **68** | 3142.38 | 2667.67 | 15875.51 |
| 7 | 0.01 | 2061.28 | 6137.16 | **38** | 27616.42 | 2448.60 | 14623.39 | **69** | 2199.98 | 2710.73 | 15729.28 |
| 8 | 0.05 | 2061.28 | 6458.54 | **39** | 27987.53 | 2461.79 | 14559.78 | **70** | 1567.81 | 2764.27 | 15561.55 |
| 9 | 0.36 | 2061.28 | 6791.50 | **40** | 28141.54 | 2476.24 | 14436.74 | **71** | 1160.54 | 2819.72 | 15331.33 |
| 10 | 2.13 | 2061.30 | 7138.35 | **41** | 28140.52 | 2488.22 | 14293.93 | **72** | 877.11 | 2877.54 | 15035.33 |
| 11 | 10.37 | 2061.43 | 7514.60 | **42** | 28154.92 | 2494.85 | 14192.26 | **73** | 657.06 | 2947.47 | 14720.53 |
| 12 | 41.53 | 2062.06 | 7941.17 | **43** | 28286.21 | 2495.79 | 14193.49 | **74** | 480.53 | 3042.13 | 14444.88 |
| 13 | 138.09 | 2064.57 | 8420.26 | **44** | 28532.48 | 2492.67 | 14318.64 | **75** | 348.61 | 3180.63 | 14220.90 |
| 14 | 383.46 | 2072.34 | 8919.37 | **45** | 28876.84 | 2488.02 | 14534.76 | **76** | 265.63 | 3391.66 | 14002.24 |
| 15 | 896.72 | 2090.85 | 9393.66 | **46** | 29308.15 | 2484.69 | 14779.67 | **77** | 224.24 | 3662.37 | 13757.15 |
| 16 | 1784.90 | 2124.99 | 9821.66 | **47** | 29736.85 | 2485.35 | 14993.73 | **78** | 205.05 | 3912.63 | 13529.21 |
| 17 | 3058.760 | 2173.19 | 10208.13 | **48** | 29979.63 | 2491.48 | 15137.77 | **79** | 194.11 | 4096.57 | 13361.83 |
| 18 | 4595.07 | 2224.39 | 10569.01 | **49** | 29899.97 | 2502.72 | 15202.24 | **80** | 187.08 | 4230.83 | 13254.00 |
| 19 | 6253.920 | 2265.13 | 10931.49 | **50** | 29532.39 | 2516.79 | 15211.28 | **81** | 182.78 | 4345.39 | 13189.57 |
| 20 | 8036.37 | 2289.99 | 11332.01 | **51** | 29026.81 | 2530.41 | 15210.36 | **82** | 180.45 | 4461.27 | 13154.38 |
| 21 | 10057.18 | 2302.23 | 11793.20 | **52** | 28470.50 | 2541.16 | 15240.14 | **83** | 179.36 | 4589.10 | 13137.61 |
| 22 | 12379.79 | 2307.66 | 12300.34 | **53** | 27787.97 | 2548.81 | 15320.06 | **84** | 178.95 | 4731.27 | 13130.99 |
| 23 | 14894.86 | 2310.64 | 12806.16 | **54** | 26834.23 | 2554.67 | 15454.76 | **85** | 178.82 | 4884.53 | 13128.90 |
| 24 | 17341.51 | 2313.30 | 13269.43 | **55** | 25565.05 | 2560.09 | 15647.00 | **86** | 178.79 | 5041.78 | 13128.38 |
| 25 | 19462.06 | 2316.29 | 13685.66 | **56** | 24076.93 | 2566.02 | 15897.90 | **87** | 178.78 | 5192.97 | 13128.28 |
| 26 | 21145.37 | 2320.10 | 14064.38 | **57** | 22464.91 | 2573.80 | 16189.60 | **88** | 178.78 | 5327.44 | 13128.27 |
| 27 | 22428.04 | 2325.93 | 14385.32 | **58** | 20691.33 | 2585.30 | 16462.90 | **89** | 178.78 | 5438.16 | 13128.26 |
| 28 | 23407.02 | 2335.50 | 14595.22 | **59** | 18670.64 | 2601.24 | 16628.30 | **90** | 178.78 | 5523.91 | 13128.26 |
| 29 | 24176.54 | 2349.71 | 14648.03 | **60** | 16466.82 | 2618.98 | 16637.84 |  |  |  |  |
| 30 | 24813.30 | 2367.22 | 14546.20 | 61 | 14301.00 | 2633.56 | 16546.92 |  |  |  |  |

GDP, gross domestic product; UK, United Kingdom

*3.4.5. Parameters of the utility function*

Table S1.8 summarizes the values we use to calibrate the utility-function-related parameters in $\left( 11 \right), \left( 12 \right),$ and $(15)$ and the budget-constraint-related parameters in $(16)$ and $(17)$. We use the value of $r$ (interest/discount rates) both in the lifecycle model and in the discounting of quality adjusted life years ($QALY$) given by equations (49a)- (49c) below.

**Table S1.8*.* Utility function and budget-constraint-related parameter values**

|  | UK | Calculation |
| --- | --- | --- |
| $\boldsymbol{\sigma}$ | 1.3551 |  |
| $\boldsymbol{c}_{\boldsymbol{0}}$ (GBP, 2018) | $619.8900$ | $4.24\times365.25\times0.706052\times105.9/93.4/2=619.88604$ |
| $\boldsymbol{\alpha}$ | 0.2227 |  |
| $\boldsymbol{a}_{\boldsymbol{c}}$ | 0.7730 |  |
| $\boldsymbol{\rho}$ | 0.0350 |  |
| $\boldsymbol{r}$ | 0.0350 |  |
| $\boldsymbol{T}$ | 4698.2546 (12.836312/day) |  |
| $\boldsymbol{A}$ | 86753.9600 |  |

A initial wealth; $\alpha$ elasticity of substitution between goods and services and non-market time; $a_{c}$ elasticity of substitution between goods and services and consumption; $c_{0}$ consumption*;* $\sigma$ elasticity of intertemporal substitution; $\rho$ utility interest/discount rate;$r$ interest/discount rate; GBP British pound sterling; T annual time endowment; UK United Kingdom

Age-specific health utilities in the uninfected state is denoted by $q_{u}=(q_{u}\left( 0 \right)\ldots q_{u}\left( 100 \right))$ and health utility in the given health state expressed as a fraction of health utility in the uninfected state is denoted by $q^{scale}=(0,1,q_{3}^{scale},\ldots,q_{19}^{scale})$. The QALYs without vaccination is given by:

$$\begin{aligned} {QALY}^{nv}=\sum_{i=a}^{100} \frac{q_{u}\left( i \right)*q^{scale^{'}}*s^{nv}(i)}{\left( 1+r \right)^{i-a}}\#\left( 49a \right) \end{aligned}$$

The QALYs with vaccination is given by:

$$\begin{aligned} {QALY}^{v}=\sum_{i=a}^{100} \frac{q_{u}\left( i \right)*q^{scale^{'}}*s^{v}(i)}{\left( 1+r \right)^{i-a}}\#\left( 49b \right) \end{aligned}$$

The impact of vaccination on QALYs is simply the difference

$$\begin{aligned} \Delta QALY={QALY}^{v}-{QALY}^{nv}\#\left( 49c \right) \end{aligned}$$

*Calibrating* $\alpha,a_{c}$

By rearranging equation $\left( 22 \right)$ for the uninfected state, replacing model variables with data (e.g., replacing $c_{u}^{F}(i)$ with $\hat{c}_{u}^{F}\left( i \right)$, etc.), taking natural logs, and recalling that $a_{l}=1-a_{c}$, we get:

$$\begin{aligned} \ln\left( \frac{\hat{c}_{u}^{F}\left( i \right)}{\hat{c}_{u}\left( i \right)}-1 \right)=\ln\left( \frac{1-a_{c}}{a_{c}} \right)+\left( 1-\alpha\right)*ln\left( \hat{w}_{u}\left( i \right) \right).\#\left( 50 \right) \end{aligned}$$

Applying ordinary least squares (OLS) regression on our data for ages 16–64, we obtain the intercept in this regression as $\ln\left( \frac{{1-a}_{c}}{a_{c}} \right)$ and the slope as $\left( 1-\alpha\right)$, yielding estimates for $\alpha$ and $a_{c}$.

*Calibrating* $c_{0}$ *and* $z_{0}$

We take $c_{0}$ to be half of the annual extreme poverty rate as computed by Allen (2017)^20^ of $4.24 in 2011 international dollars (I$). We translate the UK value to 2018 GBP using the 2011 price purchasing power (PPP) exchange rate, inflate to 2018 using the UK CPI (Table S1.4) and annualize and divide by half to get $c_{0}=619.89$ in 2018 GBP (Table S1.8).

We assume that $l_{0}$ and $c_{0}$ jointly satisfy the optimality conditions of the model. Taking ratios of $(19)$ and $(20)$, and evaluating at $l_{0}$ and $c_{0}$, we get:

$$\begin{aligned} l_{0}=\frac{a_{l}}{a_{c}}w^{-\alpha}c_{0}.\#\left( 51 \right) \end{aligned}$$

Plugging this into $(12)$ we get:

$$\begin{aligned} \begin{aligned} z_{0}={c_{0}*\left( {a_{c}}^{\frac{1}{\alpha}}+a_{l}{*a_{c}}^{\frac{1-\alpha}{\alpha}}w^{1-\alpha} \right)}^{\frac{\alpha}{\alpha-1}} . \end{aligned}\#\left( 52 \right) \end{aligned}$$

We evaluated this expression using the lowest hourly wage: 4.98 GBP (Table S1.5).

*Calibrating* $\sigma$ *using the Value of a Statistical Life (VSL)*

We calibrate sigma by combining our expression for ${VSLY}_{u}(i)$ with empirical estimates of the VSL. More specifically, if we denote by $a_{h,}$ the integer age that was closest to half a country’s life expectancy, we compute the $\sigma$ that satisfies:

$$\begin{aligned} VSL=\sum_{i=a_{h}}^{100} \frac{\frac{l_{i}}{l_{a_{h}}}}{{(1+r)}^{i-a_{h}}}*{VSLY}_{u}\left( i \right)=\sum_{i=a_{h}}^{100} \frac{\frac{l_{i}}{l_{a_{h}}}}{{(1+r)}^{i-a_{h}}}*\left[ \hat{y}_{u}^{F}\left( i \right)+\hat{c}_{u}^{F}\left( i \right)*\frac{1-\sigma\left( \frac{z_{0}}{\hat{z}_{u}\left( i \right)} \right)^{1-\frac{1}{\sigma}}}{\sigma-1} \right]\#\left( 53 \right) \end{aligned}$$

We use the 2017 life expectancy at birth of 81.16 for the UK from the World Development Indicators,^21^ which yield the closest integer value for $a_{h}$ of 41. The ratio $\frac{l_{i}}{l_{a_{h}}}$ gives the survival rate, i.e., the percentage of a cohort alive at age $a_{h}$ that is still alive at age $i$.

We use a VSL of $4.5M in 2005 United State Dollars (USD), which is the upper range for VSL given in the Organisation for Economic Co-operation and Development (OECD) (2012) as reported in Robinson et al. (2017).^21-23^ This corresponded to 4.5*0.549998*105.9/78.1=3.356M 2018 GBP. In the sensitivity analysis, we use $3.6M in 2005 USD, as proposed for EU-27 countries by an OECD meta-analysis.^23^ We inflate this using the 2005 market exchange rate and the UK CPI: $3.6M*0.549998*105.9/78.1=2.68M in 2018 GBP.

*Calibrating* $A(a)$

We compute $A(a)$ as:

$$\begin{aligned} \hat{A}\left( a \right)=\sum_{i=a}^{100} \frac{\left[ \hat{c}_{u}\left( i \right)-\hat{y}_{u}\left( i \right) \right]s\left( i \right)}{\left( 1+r \right)^{i-a}}\#\left( 54 \right) \end{aligned}$$

*Calibrating* $T$

We take $T$ to be the arithmetic mean of $\frac{\hat{y}_{u}^{F}\left( i \right)}{\hat{w}_{u}\left( i \right)}$ across ages 16 to 64.

*3.4.6. Impact of disability on hourly wages, earnings, and hours worked*

*Disability and hourly earnings: UK*

Table B6 in Longhi (2017)^10^ shows the impact of various disabilities on hourly wages for men and women. We take the impact of a disability on the hourly wage to be the arithmetic mean of the impact on men’s and women’s wages as shown in columns (1) and (3) in this table. We depart from Longhi (2017) only in assuming that skin scarring has no effect on wages, earnings, or hours. The results of this calculation are shown in the “wage gap” columns of Table 1.

We take hours worked conditional on disability status to equal the product of the probability of employment conditional on disability status (taken from Table 2a of the supporting tables for the Work, Health, and Disability Green paper) and hours worked among the employed conditional on disability status (from Tables B1 and B2 in Longhi (2017)).^10^

Earnings conditional on disability status equal the product of hours worked conditional on disability status and hourly wages conditional on disability status. The “wage gap,” “earnings gap,” and “hours worked gap” in Table 1 are simply the ratios of wages, earnings, and hours worked, respectively, among those with the relevant disability to those without disability.

Meyer and Mok (2019)^9^ provide US-based evidence that the percentage decline in consumption resulting from disability is roughly one-third of the earnings decline from disability. More specifically, they find in a sample of household heads that, ten years after the onset of a disability, earnings of those with a chronic non-severe disability are 30% lower than earnings of those with no disability, while earnings of those with a chronic severe disability are 77% lower than earnings of those with no disability. They also find that ten years after disability onset, the sum of food and housing consumption among those with a chronic non-severe (severe) disability is 8.58% (25%) lower than the sum among those with no disability. Thus, food and housing consumption losses are slightly under one-third of earnings losses.^9^ We compute the “consumption gap” column so that the consumption shortfall is one-third of the earnings shortfall implied by the “earnings gap” column.

***3.5. Costs***

In a scenario analysis (Scenario F), we incorporate certain novel cost categories from Beck et al. (2021),^24^ as discussed below in respective sections.

*3.5.1. Averted acute phase treatment costs (*$aatc)$

Acute phase costs occur upon infection with invasive meningococcal disease (IMD). These costs, presented in Table S1.10a, are age-specific and only apply to states representing the year in which infection occurs, i.e., not long-term (year 2+) disability states. We also assume that someone suffering from a temporary disability who subsequently suffers from a long-term disability in the next year incurs the acute IMD cost twice, once per infection.

**Table S1.10a. Acute phase treatment costs^24^**

| Age | Cost (GBP) |
| --- | --- |
| 0 | 8,759.91 |
| 1–4 | 8,691.99 |
| 5–14 | 8,951.80 |
| 15–19 | 8,988.41 |
| 20–24 | 9,179.77 |
| 25–44 | 9,222.19 |
| 45–64 | 9,020.89 |
| ≥65 | 8,487.30 |

GBP British Pound Sterling

*3.5.2. Averted long-term treatment costs (*$alttc$*)*

Long-term treatment costs are annual costs associated with managing long-term disabilities. These costs are sequelae-specific but not age-specific and presented in Table S1.10b.

**Table S1.10b. Long-term treatment costs^24^**

| Sequelae | 1^st^ Year (GBP) | 2^nd^ Year Onward (GBP) |
| --- | --- | --- |
| Amputation | 22,231.75 | 1,468.80 |
| Skin scarring | 2,654.74 | - |
| Renal dysfunction/Failure/Insufficiency | 2,060.59 | 927.75 |
| Blindness/Severe vision impairment | 4,288.15 | 4,288.15 |
| Severe hearing loss | 52,165.87 | 5,301.54 |
| Moderate hearing loss | 1,517.44 | 270.34 |
| Unilateral hearing loss/Impairment | 406.86 | 200.73 |
| Epilepsy/Seizures | 2,329.79 | 2,379.79 |
| Severe neurological disorders | 4,449.97 | 917.49 |
| Speech or communication problems | 4,553.82 | - |
| Intellectual disabilities | 7,211.97 | 7,211.97 |
| Motor deficits | 5,648.94 | 3,389.37 |
| Depression | 133.63 | 133.63 |
| Anxiety | 133.63 | 133.63 |
| Separation anxiety | 133.63 | 133.63 |
| ADHD | 1,416.88 | 1,416.88 |

ADHD, attention deficit hyperactivity disorder; GBP British Pound Sterling

*3.5.3. Averted long-term formal care costs (*$alfcc$*)*

A severe disability sometimes requires ongoing formal care for the rest of the patient’s life. The annual cost of this care is assumed to be £9,136.^24^ Following Beck et al. (2021), we classified the following sequelae as severe disabilities:

- Amputation
- Blindness/severe visual impairment
- Severe hearing loss
- Severe neurological disorders
- Intellectual disabilities
- Motor deficits

Since the Markov model has two states for each of these sequelae (year 1 and years 2+), we applied formal long-term care costs to both states, representing a lifetime of care, and assumed that only 6.8% of patients with a severe disability need formal long-term care.^24^ Therefore, the expected annual cost for these patients is 0.068*£9136=£621.248.

*3.5.4. Averted litigation costs (*$alc$*)*

Consistent with Beck et al. (2021), we applied litigation costs to infections leading to severe disabilities. Since the Markov model has two states for each of these sequelae (year 1 and years 2+), we applied costs only to the year 1 state to prevent double counting.

The cost (and likelihood) of childhood (≤19) and adult (≥20) claims are £1,099,424 (1.8%) and £357,165 (4%), respectively. Payments take place over twenty years and start two years after the claim. We assume that claims are filed immediately upon infection. We treat claim payments as deferred ordinary annuities (payment at the end of each year). The present value of such an annuity is given by the following formula:

$$\begin{aligned} \text{PV}=p\left( \frac{1-\left( 1+r \right)^{-n}}{r\left( 1+r \right)^{t}} \right),\#\left( 55 \right) \end{aligned}$$

where $p$ is the payment per period, $r$ is the interest/discount rate, $n$ is the number of payment periods, and $t$ is the time (in periods) until the first payment.

The present value of child and adult claims are therefore £729,326.58 and £236,933.09, respectively. Factoring in the claim likelihoods gives us the expected present values of £13,127.88 and £9,477.32 for child and adult claims.

*3.5.5. Averted special education costs (*$asec$*)*

Annual special education costs apply for ages 0–16 in selected sequelae in both their year one and long-term states. Students suffering from blindness, severe neurological disorders, intellectual disabilities, or motor deficits incur a yearly cost of £17,840.05. Students with severe hearing loss, speech impediments, or ADHD incur annual costs of £4,401.30. Students with depression, anxiety, or separation anxiety incur annual costs of £1,195.27.

*3.5.6. Averted outbreak costs (*$aoc$*)*

Some IMD infections stem from outbreaks and therefore have public health costs, where the costs for childhood (≤19) and adult (≥20) infections are £466.25 and £437.19, respectively. Only 2.5% of infections stem from an outbreak, so we multiplied these costs by 0.025 to get expected costs per infection. As with litigation costs, public health costs only apply to states representing the first year of infection/disability.

*3.5.7. Averted cost estimates*

The averted costs are summarized in Table S1.10c as the difference between no vaccinated and vaccinated treatment arms.

**Table S1.10c Averted costs (GBP)^24^**

| Cost category | Not vaccination | Vaccinated | Averted costs |
| --- | --- | --- | --- |
| Acute phase treatment costs | 9.26 | 5.93 | 3.33 |
| Long-term treatment costs | 17.21 | 10.85 | 6.36 |
| Long-term formal caregiving costs | 1.26 | 0.79 | 0.47 |
| Litigation costs | 0.97 | 0.61 | 0.36 |
| Special education costs | 13.93 | 7.62 | 6.30 |
| Outbreak costs | 0.01 | 0.01 | 0.00 |

GBP British Pound Sterling

**References**

1. Office for National Statistics (ONS). National Life Tables, Great Britain, 1980-82 to 2013-15. <https://www.ons.gov.uk/file?uri=/peoplepopulationandcommunity/birthsdeathsandmarriages/lifeexpectancies/datasets/nationallifetablesgreatbritainreferencetables/current/nltgb1315reg.xls>. Accessed 4 May 2020.
2. Public Health England. Invasive meningococcal infections laboratory reports in England by capsular group, age group & calendar year, 2000-2014. [https://web.archive.org/web/20151013112030/https://www.gov.uk/government/uploads/system/uploads/attachment_data/file/432895/Table_9_Invasive_meningococcal_infections_lab_reports__England_by_capsular_group___age.pdf. Accessed 26 June 2018](https://web.archive.org/web/20151013112030/https://www.gov.uk/government/uploads/system/uploads/attachment_data/file/432895/Table_9_Invasive_meningococcal_infections_lab_reports__England_by_capsular_group___age.pdf.%20Accessed%2026%20June%20%202018)
3. ECDC. Atlas, Invasive Meningococcal Disease, Confirmed cases, Serogroup B notification rate, 2014, age-specific rate (Serogroup B cases). <https://atlas.ecdc.europa.eu/public/index.aspx>. Accessed 27 May 27 2016
4. Ladhani SN, Giuliani MM, Biolchi A, et al. Effectiveness of Meningococcal B Vaccine against Endemic Hypervirulent *Neisseria meningitidis* W Strain, England. *Emerg Infect Dis*. 2016;22(2):309-11.
5. Shigematsu M, Davison KL, Charlett A, Crowcroft NS. National enhanced surveillance of meningococcal disease in England, Wales and Northern Ireland, January 1999-June 2001. *Epidemiol Infect*. 2002;129(3):459-70
6. Martinon-Torres F, Safadi MAP, Martinez AC, et al. Reduced schedules of 4CMenB vaccine in infants and catch-up series in children: Immunogenicity and safety results from a randomised open-label phase 3b trial. *Vaccine*. 2017;35(28):3548-57.
7. Martinon-Torres F, Carmona Martinez A, Simkó R, et al. Antibody persistence and booster responses 24-36 months after different 4CMenB vaccination schedules in infants and children: A randomised trial. *J Infect*. 2018;76(3):258-69.
8. Murphy, M. and Topel, R. The value of health and longevity. *Journal of Political Economy*. 2006;114(5): 871-904.
9. Meyer BD, Mok WKC. Disability, earnings, and consumption. *Journal of Public Economics*. 2019;17: 51-69.
10. Longhi S. *The disability pay gap*. *Research* report 107. Equality and Human Rights Commission, 2017. <https://www.equalityhumanrights.com/sites/default/files/research-report-107-the-disability-pay-gap.pdf>. Accessed 4 May 2020
11. HM Treasury. The green book: central government guidance on appraisal and evaluation. <https://assets.publishing.service.gov.uk/government/uploads/system/uploads/attachment_data/file/938046/The_Green_Book_2020.pdf>. Accessed 27 January 2018
12. Al-Janabi H, Van Exel J, Brouwer W, et al. Measuring health spillovers for economic evaluation: a case study in meningitis. *Health Econ*. 2016;25(12):1529–1544. doi: 10.1002/hec.3259
13. ONS. Births by parents’ characteristics in England and Wales: 2015 <https://www.ons.gov.uk/peoplepopulationandcommunity/birthsdeathsandmarriages/livebirths/bulletins/birthsbyparentscharacteristicsinenglandandwales/2015>. Accessed 4 May 2016
14. Kennedy ITR, van Hoek AJ, Ribeiro S, et al. Short-term changes in the health state of children with group B meningococcal disease: A prospective, national cohort study. *PLoS One*. 2017;12(5):e0177082.
15. Christensen H, Trotter CL, Hickman M, Edmunds WJ. Re-evaluating cost effectiveness of universal meningitis vaccination (Bexsero) in England: modelling study. *BMJ*. 2014;349:g5725
16. Kind P, Hardman G, Macran S. *UK Population Norms for EQ-5D.* *Discussion paper 172.* The University of York, Centre of Health Economics, 1999. <https://www.york.ac.uk/che/pdf/DP172.pdf>. Accessed 27 May 2016
17. ONS. Consumer price inflation time series (MM23) <https://www.ons.gov.uk/economy/inflationandpriceindices/timeseries/d7bt/mm23> Accessed 4 May 2020
18. UK from the Annual Survey of Hours and Earnings. Median hourly earnings excluding overtime, provisional 2018 estimates. <https://www.ons.gov.uk/file?uri=/employmentandlabourmarket/peopleinwork/earningsandworkinghours/datasets/ashe1997to2015selectedestimates/current/ashe19972018timeseries.xls>. Accessed 4 May 2016.
19. National Transfer Accounts Project. Labor income <https://ntaccounts.org/web/nta/show/Documents/Labor%20Income> Accessed 4 May 2016.
20. Allen RC. Absolute Poverty: When Necessity Displaces Desire. *American Economic Review*. 2017;107:3690-721.
21. World Data Bank. World Development Indicators. https://databank.worldbank.org/source/world-development-indicators Accessed 4 May 2016.
22. Robinson LA, Hammitt JK, Chang AY, Resch S. Understanding and improving the one and three times GDP per capita cost -effectiveness thresholds. Health Policy and Plan. 2017;32(1):141-145.
23. OECD. Mortality risk valuation in environment, health, and transport policies. 2012. <https://www.oecd.org/env/tools-evaluation/mortalityriskvaluationinenvironmenthealthandtransportpolicies.htm>; Accessed 4 May 2016
24. Beck E, Klint J, Neine M., Garcia S, Meszaros K. Cost-Effectiveness of 4CMenB Infant Vaccination in England: A Comprehensive Valuation Considering the Broad Impact of Serogroup B Invasive Meningococcal Disease. *Value Health*. 2021;24(1):91-104
